# GH-resistant (Laron) mice: gene therapy with a liver-specific GH receptor causes unbalanced upregulation of female-biased and growth-related genes

**DOI:** 10.3389/fendo.2026.1808977

**Published:** 2026-05-28

**Authors:** Joshua K. Tay, Kian Chuan Sia, Siti Humairah Mohd Rodhi, Shu Uin Gan, Zhen Ying Fu, Maxim Pyatkov, John J. Kopchick, Michael J. Waters, David J. Waxman, Kok-Onn Lee

**Affiliations:** 1 Department of Otolaryngology-Head & Neck Surgery, National University of Singapore, Singapore, Singapore; 2 Synthetic Biology Translational Research Programme, National University of Singapore, Singapore, Singapore; 3 Department of Surgery, National University of Singapore, Singapore, Singapore; 4 Department of Biology and Bioinformatics Program, Boston University, Boston, MA, United States; 5 Institute of Molecular Medicine and Aging, Ohio University, Athens, OH, United States; 6 Institute for Molecular Bioscience, The University of Queensland, Brisbane, QLD, Australia; 7 Department of Medicine, National University of Singapore, Singapore, Singapore

**Keywords:** adeno-associated virus (AAV), differential gene expression, growth hormone receptor (GHR), IGF-1 signaling, Laron syndrome, oncogenes, RNA-Seq, sex-biased genes

## Abstract

Growth hormone (GH) receptor (GHR) mutations give rise to GH-resistance (Laron syndrome). We previously treated GH-resistant Ghr-/- mice (Laron mice) with adeno-associated virus (AAV) delivering mouse (m)Ghr controlled by a constitutively active liver-specific promoter (HLP). A single injection of AAV-HLP-mGHR resulted in a significant but limited increase in body length and weight, consistent with studies of IGF-1 treatment in humans and mice. Here, we performed RNA-seq on male and female mouse livers comprising the following groups: GHR+/+ (wild-type), GHR-/- (Laron), AAV-HLP-mGHR-treated GHR-/- (treatment group), and AAV-HLP-Luc (Luciferase)-treated GHR-/- (control group). Only four genes showed significant differential expression in GHR -/- mouse liver following Luciferase vector treatment, indicating minimal effect of the AAV-HLP vector. AAV-HLP-mGHR stimulated significant expression changes in 448 genes compared to AAV-HLP-Luc control, substantially fewer than the 2781 genes whose expression was altered in GHR-/- compared to GHR+/+. AAV-HLP-mGHR treatment induced the GH-responsive IGF signaling genes *Igf1* and *Igfals* ~16-fold compared to AAV-HLP-Luc control, but only to 40–45% of GHR+/+ liver levels. The treatment also upregulated a small subset of genes beyond GHR+/+ expression levels (p-adj < 0.05), including the proto-oncogenes *Ascl1*, *Tmprss4*, and others. Finally, genes dysregulated upon GHR loss and upregulated in livers of AAV-HLP-mGHR-treated mice were significantly enriched for sex-biased genes, consistent with the major role of GH and GHR in regulating liver sex differences. While gene replacement therapy is a potential therapy for Laron syndrome, an unregulated constitutively active promoter may drive unexpected and unbalanced changes in liver gene expression that will require monitoring.

## 1 Introduction

Gene replacement therapy aims to treat genetic disorders caused by dysfunctional genes through *in vivo* delivery of a functional genetic sequence (gene or cDNA), thereby enabling expression of a functional gene product. Several gene therapies have already been approved for clinical use (1–3). Successful gene replacement therapy requires two key elements. First, the functional gene must be efficiently delivered to the appropriate target tissue(s). Second, the therapeutic gene sequence must contain regulatory elements, such as promoter and enhancer sequences, that enable expression of the therapeutic RNA and protein in the target tissue. Gene delivery can be achieved using viral vectors, including adenovirus, adeno-associated virus (AAV), or lentivirus, or through non-viral approaches such as liposomal or polysaccharide-based vectors. In most cases, gene therapy vectors utilize constitutively active promoters that lack the complex, multi-layered regulatory mechanisms that typically govern endogenous gene expression.

Patients with growth hormone (GH) resistance caused by defects in the growth hormone receptor (GHR), a condition known as Laron syndrome, have a rare genetic disorder characterized by dwarfism and severe postnatal growth failure (4). These defects in GHR interfere with GH binding to its receptor and/or downstream signaling events (5), resulting in reduced hepatic production of insulin-like growth factor 1 (IGF1), the primary mediator of postnatal growth (6). In addition, GH regulates the cell surface levels of GHR through negative feedback mechanisms involving SOCS family proteins that target GHR for ubiquitin-dependent degradation (7, 8). This GH-mediated downregulation of GHR signaling has stimulated efforts to develop GHR superagonists with prolonged activity to partially offset receptor turnover and increase signaling output (9).

Patients with Laron syndrome are currently treated with recombinant IGF1 administered either once daily (10) or twice daily (11–13). IGF1 may also be co-administered with insulin-like growth factor–binding protein 3 (IGFBP3) to prolong its biological activity (14–16). Although these treatments stimulate increases in height and body weight, overall growth responses remain limited, even in patients who initiate therapy early in life (17, 18). Consistent with these clinical observations, we previously reported similar outcomes in a mouse model of GH resistance and IGF1 deficiency (Laron syndrome, OMIM #262500) (4), caused by functional loss of the GHR, following a single administration of an AAV vector expressing mouse GHR (AAV-HLP-mGHR) (19).

The vector used in those studies, AAV-HLP-mGHR, a highly efficient 251-bp synthetic liver-specific promoter (‘HLP promoter’) composed of a 34-bp core enhancer from the human apolipoprotein E hepatic control region and a modified 217-bp human alpha-1-antitrypsin gene promoter, in which the distal X and proximal A+B regulatory domains were joined (20, 21). This promoter has been widely employed in both preclinical (22–25) and clinical (26–28) studies for liver-targeted gene therapy. Hepatic expression of functional mGHR increased circulating levels of IGF1, IGFBP3, and the acid-labile subunit (IGFALS), resulting in significant improvements in body length (male: 5.7% increase, reaching 79% of wild-type; female: 5.1% increase, reaching 81% of wild-type). In contrast, body weight increased disproportionately (male: 12% increase, reaching 60% of wild-type; female: 11% increase, reaching 66% of wild-type), leading to an obese phenotype. These effects were observed 16 weeks after AAV administration (20–21 weeks of age) compared with mice treated with the control vector AAV-HLP-Luc encoding luciferase (19). Despite these improvements, growth restoration remained incomplete and was comparable in magnitude to that reported in GHR-deficient mice treated with IGF1 (29, 30) and in clinical studies of Laron syndrome patients receiving recombinant IGF1 therapy (17, 31).

Here, we characterized the broader transcriptional impact of GHR gene therapy using AAV-HLP-mGHR. We analyzed whole-liver transcriptomes from livers obtained in our previous study and compared gene expression profiles between mice treated with AAV-HLP-mGHR and control mice treated with AAV-HLP-Luc (19). Our results demonstrate that AAV-HLP-mGHR gene therapy partially reverses the molecular consequences of GHR deficiency. Notably, we observed sex-specific differences in gene expression responses to both GHR loss and AAV-HLP-mGHR treatment, highlighting the complex sexually dimorphic effects of GH signaling on liver transcriptional programs (32–34). Unexpectedly, AAV-HLP-mGHR also induced expression of genes associated with cell proliferation and cancer. These findings underscore the potential risks of employing constitutively active, unregulated promoters in GHR gene therapy and provide important insights into both efficacy and safety considerations of this approach.

## 2 Materials and methods

### 2.1 Animal studies

Animal studies were performed previously in accordance with guidelines and protocols approved by the Institutional Animal Care and Use Committee (IACUC) of the National University of Singapore (protocol nos. BR18–0735 and R18-0750) (19). Briefly, 4–5-week-old male and female Laron dwarf mice were treated with 4 × 10¹^0^ vector genomes per mouse (vg/mouse) of AAV-HLP-mGHR (single-stranded AAV serotype 8) via intraperitoneal injection. A control group of Laron dwarf mice received an equal dose of AAV-HLP-Luc (single-stranded AAV serotype 8), serving as a negative vector control. At 25–26 weeks post-injection (corresponding to 30–31 weeks of age), the AAV-treated Laron mice were euthanized and liver tissues were immediately collected in RNAlater (Ambion, Austin, TX, USA) for preservation and stored at −80 °C. Uninjected control mice (i.e., GHR+/+ and GHR-/- groups) were euthanized at 21 weeks of age (corresponding to 16 weeks post-injection in the treated groups).

### 2.2 RNA sequencing

Total RNA was extracted from livers of GHR+/+ (wild-type) mice, GHR-/- (Laron) mice, and from Laron mice treated with AAV-HLP-Luc (vector control) or AAV-HLP-mGHR (19). RNA from three male and three female livers from each group were analyzed. RNA-Seq was performed at Novogene-AIT Genomics, Singapore. Briefly, total liver RNA was extracted using AllPrep DNA/RNA Mini Kit (QIAGEN, Valencia, CA, USA) and polyA-RNA was then isolated using oligo-dT magnetic beads. After fragmentation, first-strand cDNA was synthesized using random hexamer primers and second-strand cDNA was synthesized using dUTP to obtain a directional (stranded) sequencing library after end repair, A-tailing, adapter ligation, size selection, enzymatic digestion, amplification, and purification. Libraries were quantified using a Qubit instrument, real-time PCR and Bioanalyzer analysis for insert size distribution. Libraries were pooled and sequenced on an Illumina NovaSeq 6000 instrument at Novogene-AIT Genomics, Singapore.

### 2.3 Mapping and quantification of gene expression

Mapping and quantification of gene expression were performed by the sequencing provider. Raw sequencing reads were first subjected to quality control and processed into clean reads using fastp (version 0.23.1), including the removal of reads with adapter contamination, reads with high content of uncertain nucleotides (N > 10%) and reads with low-quality nucleotides (base quality less than 5 constituting more than 50% of the read). Clean reads were then mapped to the mm39 mouse genome (Ensembl release 107) using Hisat2 v2.0.5 (35). FeatureCounts v1.5.0-p3 (36) was used to count reads mapping to each gene, and FPKM values of each gene (Fragments Per Kilobase of transcript sequence per Million mapped reads) were calculated based on gene length and read counts mapping to the gene.

### 2.4 Differential gene expression and pathway analysis

Downstream analysis was performed in R (v4.1.0). Principal component analysis (PCA) was performed to assess consistency of biological replicates and relationships between treatment groups. PCA was performed by filtering for highly expressed genes (FPKM > 5), followed by singular value decomposition and plotting the top two principal components.

Differential gene expression analysis was performed using DESeq2 (v1.32.0) (37). In the primary analysis, DESeq2 models were specified with a design of ~ treatment, pooling male and female samples within each group. Sex was not included as a covariate given our objective to identify treatment-responsive genes averaged across both sexes, rather than to estimate treatment × sex interactions. Differentially expressed genes were identified using the default Wald test with a false discovery rate (FDR) threshold of 0.05, without applying a fold-change cutoff. The input for DESeq2 analysis was a matrix of raw gene counts, as DESeq2 does not require normalized input for its statistical framework. To assess sex-related differences directly, a separate analysis was performed comparing male and female mice within each biological condition, thereby characterizing sex-biased expression and responses alongside the treatment effects. Heatmaps were generated using the pheatmap package (v1.0.12), based on log2-transformed FPKM values that were subsequently standardized to Z-scores by centering and scaling each gene.

Gene set enrichment analysis (GSEA) was performed using the package Fgsea (1.18.0) (38), with Hallmark and GO gene sets downloaded from the Molecular Signatures Database v2023.2 (Broad Institute) (39). The lists of differentially expressed genes and pathways are provided in the Supplementary Materials accompanying the manuscript.

### 2.5 Immunohistochemistry

Liver tissue excised from euthanized mice was fixed in 4% formaldehyde and embedded in paraffin. Deparaffinized slides were incubated with a 1:50 dilution of rabbit anti-mouse GHR antibody (Sinobiological, Beijing, China) at 4 °C overnight. Staining was performed using UltraVision Quanto Detection System HRP DAB (Thermo Fisher Scientific).

### 2.6 Statistical analyses

For genomic analyses, statistical significance was reported using the *adjusted p*-value determined by DESeq2 analysis, with an *adjusted p*-value of less than 0.05 considered statistically significant. For non-genomic analyses involving two groups, statistical significance was determined by Student’s unpaired *t*-test; values are expressed as mean ± SD. A *p*-value less than 0.05 was considered statistically significant, where * indicates *p* < 0.05, ** indicates *p* < 0.01, and *** indicates *p* < 0.001.

## 3 Results

### 3.1 Gene expression changes between AAV-HLP-mGHR and AAV-HLP-Luc treated mice

**Figure 1:**
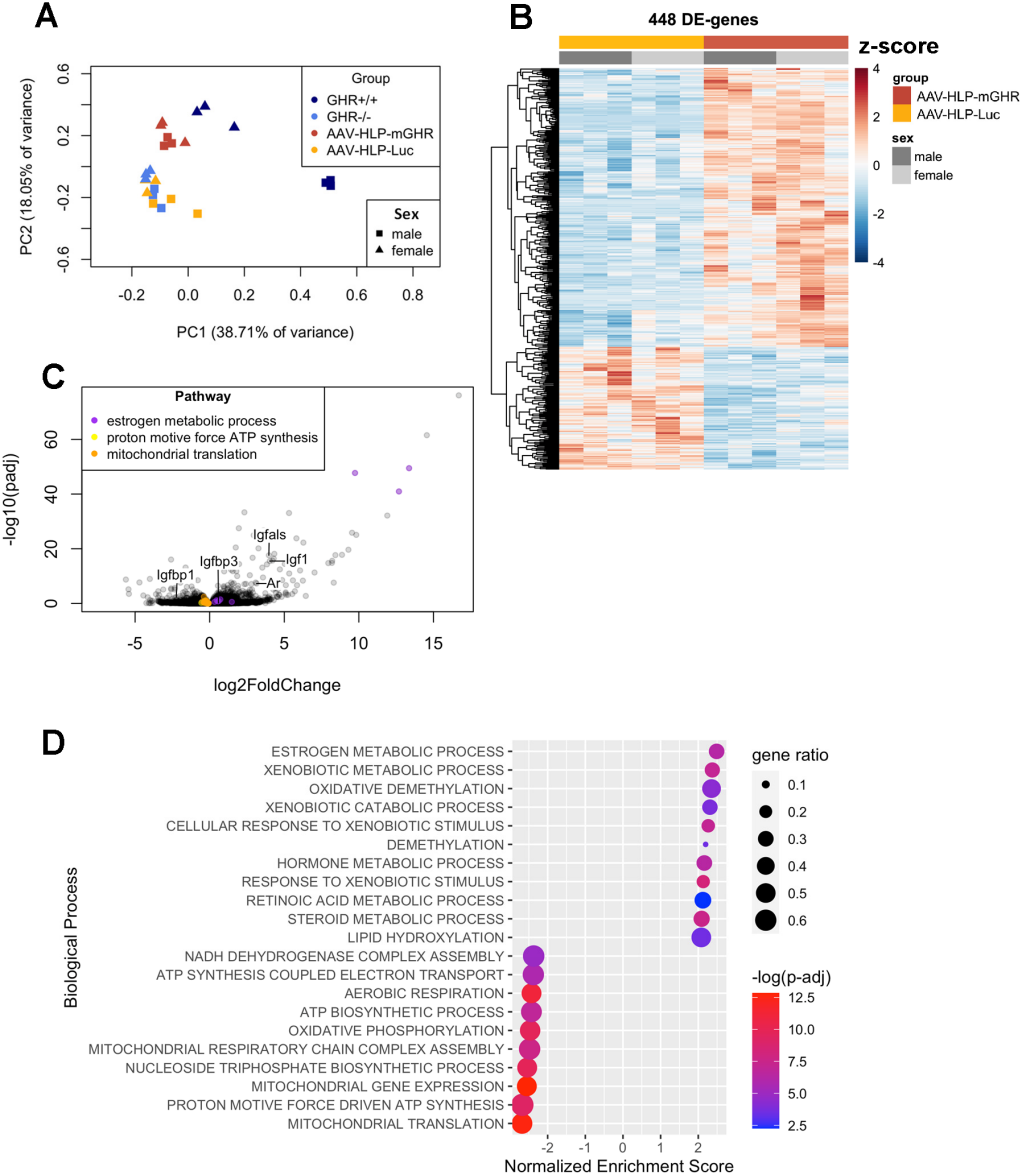
Gene expression changes between AAV-HLP-mGHR and AAV-HLP-Luc. **(A)** principal component analysis plot of gene expression libraries (n = 24). **(B)** heatmap of differentially expressed genes comparing AAV-HLP-mGHR to AAV-HLP-Luc (n = 12; male and female mice included). **(C)** volcano plot of differentially expressed genes comparing AAV-HLP-mGHR to AAV-HLP-Luc. **(D)** gene set enrichment analysis of the top biological pathways significantly enriched and significantly depleted in livers infected with AAV-HLP-mGHR compared to AAV-HLP-Luc.

RNA-seq of total liver RNA was performed to evaluate the impact of GHR loss in Laron mice and the impact of AAV-HLP-mGHR replacement therapy. Principal component analysis of the transcriptomic data revealed four distinct groups, reflecting the impact of sex and GHR phenotype (**Figure 1A**). Control and AAV-HLP-Luc-treated GHR-/- livers clustered together, while the AAV-HLP-mGHR-treated mouse livers clustered separately, reflecting changes in gene expression after AAV-HLP-mGHR rescue. Livers clustered by sex in the GHR+/+ wild-type group, but not in the GHR-/- or AAV-HLP-mGHR groups. Thus, (1) GHR loss substantially abolishes liver sex differences, consistent with prior studies indicating that GH signaling through GHR is a major determinant of sex differences in mouse liver (32, 34); and (2) a single treatment with AAV-HLP-mGHR partially restored sex-dependent patterns of liver gene expression seen in GHR-intact mice.

A total of 448 genes were differentially expressed (p-adj < 0.05) between AAV-HLP-mGHR and AAV-HLP-Luc livers when combining male and female mice in each group (**Figure 1B**). Genes upregulated in livers of AAV-HLP-mGHR mice included key genes involved in Igf signaling, namely Igf1, Igfals, Igfbp3 and Ar. AAV-HLP-mGHR treatment increased expression of the GH-responsive IGF signaling genes Igf1 and Igfals by approximately 16-fold compared with AAV-HLP-Luc controls; however, their expression reached only 40–45% of levels observed in GHR+/+ livers (**Supplementary Table 1**). In contrast, IGFBP1, which acts as an inhibitor of IGF signaling by complexing IGFs, was downregulated (**Figure 1C**). The top pathways upregulated following AAV-HLP-mGHR treatment were steroid-related processes, including estrogen and xenobiotic metabolic processes (**Figure 1D**). We also observed consistent downregulation of mitochondrial processes, including mitochondrial translation, proton motive force-driven ATP synthesis and aerobic respiration; these changes may relate to the obesity seen in the AAV-HLP-mGHR treated mice, as observed by Sia et al. (19). Importantly, we observed only four differentially expressed genes between GHR-/- and AAV-HLP-Luc livers, suggesting very limited off-target effects on gene expression from a single dose of AAV infection (**Supplementary Figure 1**).

### 3.2 Rescue effect of AAV-HLP-mGHR dose to the liver

**Figure 2:**
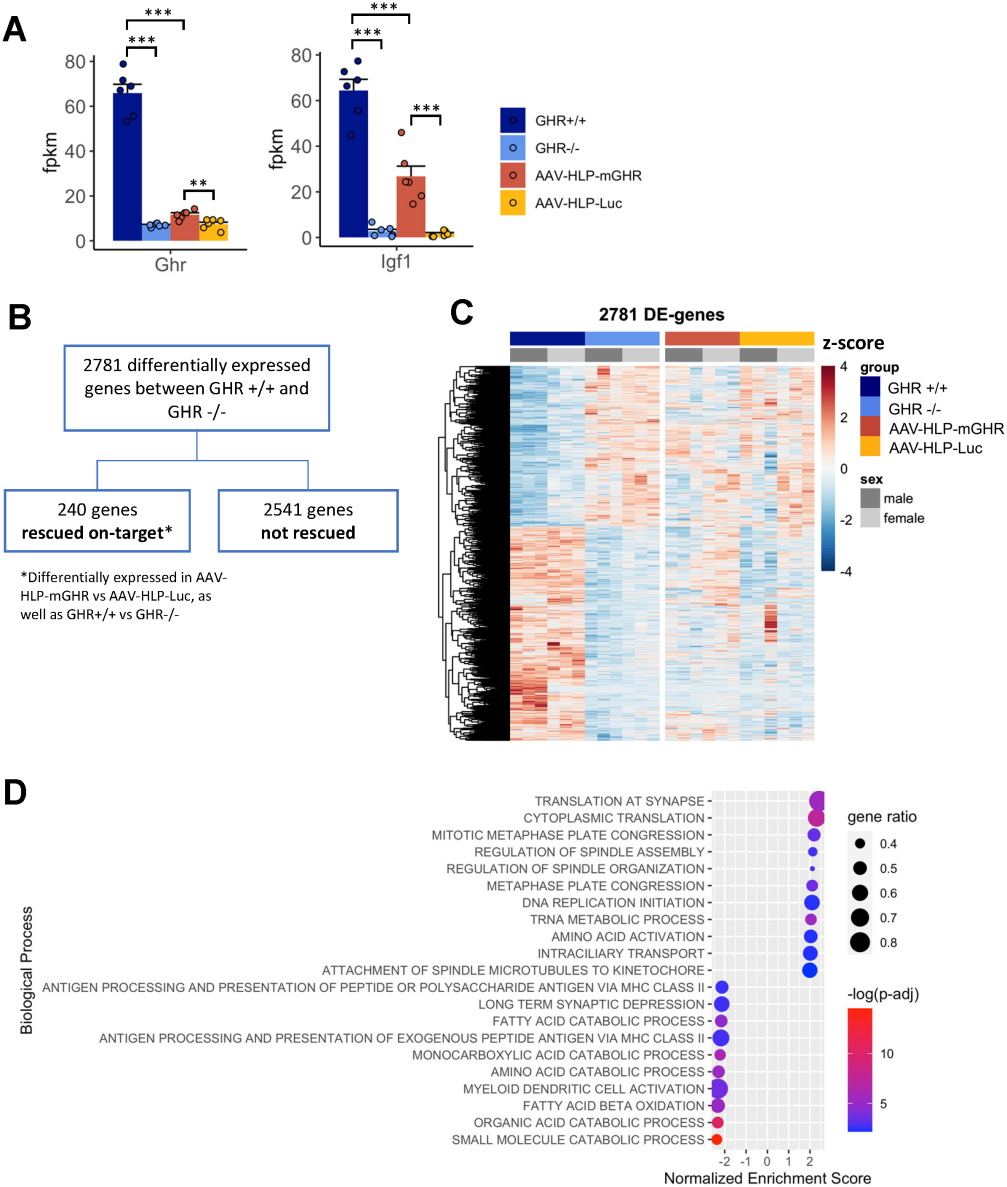
Rescue effect of a single AAV-HLP-mGHR dose to the liver. Data based on a total of n = 24 male and female mice. **(A)** Ghr and Igf1 gene expression across the four groups (n = 6; student’s unpaired t-test where *** indicates *p* < 0.001). **(B)** distribution of genes successfully rescued. **(C)** gene expression heatmap of differentially expressed genes between wild-type and knock-out mice, showing a limited number of genes successfully rescued in AAV-HLP-mGHR. **(D)** gene ontology biological pathways not rescued (enriched in wild-type compared to AAV-HLP-mGHR liver).

While Laron mice receiving a single AAV-HLP-mGHR injection showed an increase in body weight and length in our previous study (19), their growth remained reduced compared to GHR+/+ wild-type mice. We therefore evaluated the extent of rescue based on gene expression levels. *Ghr* RNA levels were increased in AAV-HLP-mGHR compared to AAV-HLP-Luc livers (mean FPKM 11.6 ± 2.2 vs 7.4 ± 2.2, p-adj < 0.01), but to a level significantly lower than found in GHR+/+ mice (mean FPKM 65.8 ± 9.7, p-adj < 0.001; **Figure 2A**). Nonetheless, despite the modest increase in GHR expression, a much more substantive increase in Igf1 expression was seen in the AAV-HLP-mGHR-treated livers, reaching 41.5% of GHR wild-type mice (mean FPKM 26.7 ± 11.1 vs 64.4 ± 12.1, p-adj < 0.001). The corresponding measurements of circulating IGF1 and related endocrine parameters, including ALS, IGFBP3, and GH were reported in our earlier study (19).

Overall, 2781 genes were differentially expressed when comparing livers from GHR+/+ and GHR-/- mice, of which 240 genes were successfully rescued in AAV-HLP-mGHR mice compared to AAV-HLP-Luc mice (**Figures 2B, C**; **Supplementary Table 1**). Gene set enrichment analysis confirmed that these on-target rescued genes were consistent with the overall gene expression changes shown in **Figure 1D**, with an increase in liver metabolic processes such as the xenobiotic metabolic process and oxidative demethylation (**Supplementary Figures 2A, B**; **Supplementary Table 1**).

Critically, key biological processes relating to cellular proliferation (DNA replication, spindle assembly and cytoplasmic translation) were not rescued by the AAV-HLP-mGHR treatment (**Figure 2D**). GHR+/+ mice also showed a decrease in small molecule catabolic processes, including fatty acid oxidation, compared to AAV-HLP-mGHR mice. Thus, AAV-HLP-mGHR mice showed a partial rescue of liver gene expression, consistent with the limited increases in body weight and length (19).

### 3.3 Increased expression of proliferation and cancer-associated genes

Apart from Igf1, we identified a list of 20 proliferation and cancer-associated genes that were upregulated in AAV-HLP-mGHR mice compared to AAV-HLP-Luc mice (**Table 1**). Nine of the top 10 upregulated proliferation and cancer-associated genes had ChIP-seq binding sites for the GH-activated liver transcription factor Stat5 in mouse liver chromatin within 10 kb (40). This finding supports the view that these genes are induced by GHR signaling directly through Stat5. The top three most highly induced genes, Ascl1 (41), Dct (42) and Prok1 (43) (>300-fold increases) are associated with small cell lung cancer (SCLC), breast cancer, glioblastoma and melanoma. These changes appear specific to the GHR transgene, as they were not observed in AAV-HLP-Luc mice compared to GHR-/- mice. For a majority of the cancer-associated genes induced in AAV-HLP-mGHR mice, expression levels were still lower than in wild-type GHR+/+ mice, consistent with the low level of Ghr expression (**Supplementary Table 2**). A notable exception was Ascl1, whose expression was undetectable in wild-type liver but showed >1000-fold upregulation in livers of AAV-HLP-mGHR-treated GHR-/- mice of both sexes.

**Table 1:** Upregulation of cancer-related genes in AAV-HLP-mGHR treated mice compared to AAV-HLP-Luc mice.

Gene (Reference)	Log2FoldChange AAV-GHR /AAV-Luc (significance, p*_adj_*)	Gene expression exceeds GHR+/+ wild-type	Association with pathology
*A1bg* (60)	+14.56 (p<0.0001)	No	Liver cancer prognostic marker, elevated in non-small cell lung cancer (NSCLC).
*Ascl1* (41)	+11.91 (p<0.0001)	Yes, both male & female	Small Cell Lung cancer. Hepatocellular carcinoma. Driver of Wnt11. Confers chemoresistance in breast cancer. Role in neuronal commitment and differentiation. Glioblastoma.
*Dct* (42)	+9.30 (p<0.0001)	No	Melanoma. Retinoblastoma. Confers radiation and chemoresistance. Melanogenic.
*Prok1* (43)	+8.32 (p<0.0001)	No	Colorectal cancer, angiogenic. Elevated in testis, prostate, ovarian, breast cancer. Central factor in human placentation with role in trophoblast invasion.
*Dntt* (61)	+5.16 (female)^*^ (p<0.0001)	No	Acute Lymphoblastic Leukemia (ALL), Chronic Myeloid Leukemia (CML). Expressed in pre-B and pre-T lymphoid cells. DNA nucleotide terminal transferase generating antigen receptor diversity.
*Cux2* (62)	+4.71 (p<0.0001)	Yes, male only	Thyroid cancer, breast cancer. Transcription factor controlling neuronal proliferation, dendrite branching and synapse formation.
*Igf1* (63)	+4.03 (p<0.0001)	No	Breast, prostate, thyroid cancer promotion
*Celsr1* (64)	+3.60 (p<0.0001)	No	Metastasis of scirrhous gastric cancer, ovarian cancer. Cadherin like, nerve formation during embryonic stage.
*Smad9* (65)	+3.21 (female)^*^ (p<0.0001)	No	Neuroblastoma. Transcriptional regulator in bone morphogenetic protein (BMP) signaling.
*Ar* (66)	+3.14 (p<0.0001)	No	Prostate cancer, breast cancer promotion.
*Tmprss4* (67)	+3.08 (p<0.0001)	Yes, male only	Thyroid carcinoma, pancreatic, breast, lung, liver, colon cancer. Transmembrane protease associated with Epithelial-Mesenchymal Transition (EMT), invasion and metastasis.
*Zap70* (68)	+2.11 (female)^*^ (p<0.002)	No	Chronic lymphocytic B-cell leukemia. Tyrosine kinase with role in T-cell development and lymphocyte activation.
*Nrep* (69)	+1.87 (p<0.0096)	No	Breast cancer, gastric cancer progression, non-alcoholic fatty liver disease (NAFLD). Neuronal regeneration, neuron differentiation in embryo and adult brain.
*Lratd2* (70)	+1.75 (p<0.0001)	Yes, male only	Glioma, breast cancer, pancreatic ductal adenocarcinoma, squamous cell carcinoma (SCC), prostate cancer. Ubiquitous expression.
*Bmper* (71)	+1.76 (female)^*^ (p<0.0068)	No	Malignant tumours of lung, colon, ovaries and uterus. Inhibits bone morphogenetic protein 2/4 (BMP2/4) action. Insulin sensitivity.
*Lamc2* (72)	+1.65 (female)^*^ (p<0.0129)	No	NSCLC. Promotes epidermal growth factor receptor (EGFR) membrane localization and biomarker for tyrosine kinase inhibitor sensitivity. Induces EMT and metastases in lung cancer cells.
*Lifr* (73)	+1.60 (p<0.0001)	No	Cancer stem cell activation. Melanoma promotion, pancreatic, lung cancer
*Wnt5b* (74)	+1.89 (female)^*^ (p<0.0001)	No	NSCLC. Instigates epithelial cancer cell invasion. Regulates chondrocyte cell stacking in growth plate.
*Wfdc2* (75)	+1.56 (p<0.0017)	No	Promotes ovarian cancer metastasis. Lung adenocarcinoma.
*Egfr* (76)	+1.41 (female)^*^ (p<0.0351)	No	Breast, lung cancer, hepatocellular cancer, liver regeneration. Receptor tyrosine kinase.
*Ajuba* (77)	+1.38 (female)^*^ (p<0.0001)	No	Colorectal cancer, head and neck squamous cell carcinoma. Regulates proliferation of skin stem cells through wingless-related integration site (Wnt) and Hippo signalling.

* Female mice only. Male mice showed no significant differences.

### 3.4 Effects of GHR-/- on genes of carbohydrate and lipid metabolism

GHR-/- mice showed increased liver expression compared to GHR+/+ mice for four key genes implicated in carbohydrate and lipid metabolism: Cd36, Pparg, Ppargc1a and Scd2. However, AAV-HLP-mGHR treatment did not restore wild-type liver expression levels of these genes, although partial recovery is evident in the principal component analysis result in **Figure 1A** and in the statistically significant difference between GHR-/- and AAV-HLP-mGHR for Ppargc1 transcripts (see **Supplementary Tables 3**, **4**). This finding is consistent with the low level of GHR expression observed in AAV-HLP-mGHR-treated mice as shown in **Figure 2A** (left panel) and was confirmed by immunohistochemistry analysis of mGHR expression (**Supplementary Figure 3**). This supports the gene set enrichment analysis shown in **Figure 1D**, which indicated downregulated mitochondrial activity resulting from AAV-HLP-mGHR treatment. It is known that GHR deficiency increases lipid uptake and oxidation (44) via increased expression of Pgc1a and Pparg. Hence decreasing uptake of free fatty acids (FFA) would be expected to decrease mitochondrial oxidative phosphorylation (ox phos) and ATP production, as evident in the GSEA. Partial lowering of Pgc1a and Pparg transcripts by mGHR is expected to facilitate decreased lipid uptake. In agreement with this view, Lpl expression decreased 9-fold in males and 3.9-fold in females following AAV-HLP-mGHR treatment.

### 3.5 Sex dependence of responses to AAV-HLP-mGHR

**Figure 3:**
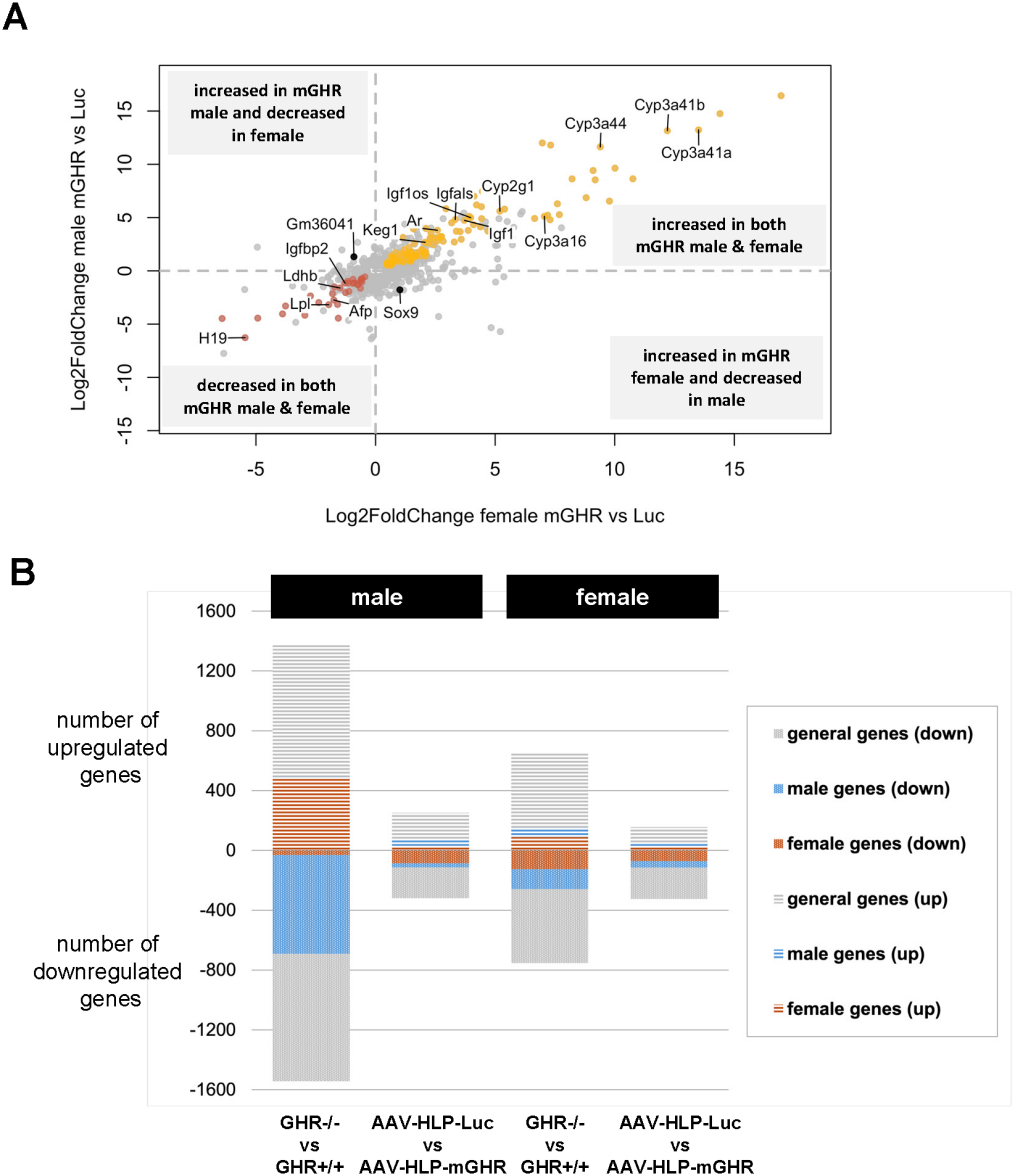
Differences between male and female rescue. **(A)** log fold-change plot of differentially expressed genes in AAV-HLP-mGHR compared to AAV-HLP-Luc, determined separately for male mice and for female mice. Statistically significant genes (p-adj < 0.05) are highlighted in yellow (upregulated in both male and female AAV-mGHR treated mice), brown (downregulated in both male and female AAV-mGHR treated mice) and black (enriched in either sex only), while gray indicates other not significant genes (p-adj >0.05). **(B)** number of differentially expressed genes (p-adj < 0.05) in GHR+/+ vs GHR-/- mice and AAV-mGHR vs AAV-Luc mice identified in each sex. Genes showing higher expression in male GHR+/+ mice than in female GHR +/+ mice (male-biased genes) are shown in blue (“male genes”), while genes showing higher expression in female GHR+/+ mice than in male GHR +/+ mice (female-biased genes) are shown in brown (“female genes”).

We observed overall consistency between the sexes in gene responses to AAV-HLP-mGHR (**Figure 3A**; also see **Figure 1B**), as seen for genes in the Igf signaling pathway (Igf1, Igfals, Igfbp3). Genes uniquely responsive to GHR in one sex included Sox9, which showed increased expression in AAV-HLP-mGHR-treated female mice, and Gm36041, which was increased in AAV-HLP-mGHR treated male mice. To further investigate the effects of AAV-HLP-mGHR treatment on genes typically expressed in liver in a sex-specific manner, we first compared gene expression between GHR+/+ male and GHR+/+ female mice and identified 1378 male-biased genes and 1203 female-biased genes (**Supplementary Material File 5A**). We then examined the impact of GHR loss (GHR+/+ vs GHR-/- livers) and of mGHR treatment (AAV-HLP-mGHR vs AAV-HLP-Luc livers) in each sex. Importantly, 479 of 1371 (35%) of the genes that were upregulated in male GHR-/- compared to male GHR+/+ liver showed female-biased expression in wild-type liver, whereas only 1% of the upregulated genes were male-biased genes. Furthermore, 662 of 1543 downregulated genes (43%) were male-biased genes, whereas only 1.9% were female-biased genes (**Figure 3B**, first bar; **Supplementary Table 5****;** **Supplementary Material Files 6A–9B****).** In contrast, loss of GHR in female mouse liver (GHR-/-) resulted in similar effects on male-biased genes as on female-biased genes (**Supplementary Table 5****;** see percentages in row 4 vs percentages in row 2). Thus, loss of hepatic GHR resulted in significantly greater disruption of sex-biased gene expression in livers of male as compared to female mice. However, only a small fraction of sex-biased gene expression was restored by AAV-HLP-mGHR treatment (**Figure 3B**, columns 2 vs 1, and columns 4 vs 3).

## 4 Discussion

In a prior study, we established and discussed the therapeutic efficacy of AAV-HLP-mGHR in a mouse model of Laron syndrome (19). We described the limitations observed in the treated mice, which were similar to some of the limitations found after treatment with IGF1 in patients. In the present study, we investigated the impact of AAV-HLP-mGHR treatment on the liver transcriptome of Laron mice, where mGHR expression in the liver is driven by a constitutively active, unregulated liver-specific promoter. Our findings highlight the limitations of *in vivo* gene replacement therapy when using a constitutively active promoter that does not recapitulate the complex regulation of the endogenous gene. AAV-HLP-mGHR−mediated expression of GHR partially restored hepatocyte GH responsiveness, as confirmed by the upregulation of IGF1 expression, as well as that of the acid-labile subunit and other IGF1-related genes. However, regulation mediated by the endogenous GHR promoter is absent, as is splicing regulation, and consequently, physiological control of hepatic GHR abundance and isoform composition, which directly impacts the generation and secretion of the mGHR splicing variant-derived GH-binding protein (45).

We observed an asymmetric impact of GHR loss on sex-biased gene expression in male liver, with many female-biased genes upregulated and many male-biased genes downregulated in GHR-/- liver. GHR loss thus recapitulates gene responses seen in male mouse liver following ablation of liver GH signaling after hypophysectomy (46), which leads to decreased expression of the many male-biased genes reliant on male plasma GH pulses and GH pulse-induced signaling via the transcription factor STAT5 (47). GHR loss also relieves inhibitory effects of the male pattern of pulsatile signaling on many female-biased genes, whose expression is derepressed in male liver upon loss of this signaling pathway (46). This sex-differential sensitivity to GHR levels, and the inability of AAV-HLP-mGHR to substantially restore sex-biased gene expression in liver has important implications for gene therapy approaches, suggesting that therapeutic strategies may need to be tailored based on biological sex to achieve optimal restoration of liver sexually dimorphic functions.

One possible explanation for incomplete restoration of sex-biased liver gene expression in AAV-HLP-mGHR–treated mice is that circulating IGF1 levels remain too low to fully re-establish normal hypothalamic–pituitary negative feedback on GH secretion. In GHR+/+ mice, liver-derived IGF1 exerts major negative feedback effects on pituitary somatotrophs and on hypothalamic GHRH/somatostatin circuits, thereby constraining overall GH output while preserving the sex-specific temporal pattern of GH secretion that underlies male- and female-biased liver transcriptional programs (48, 49). In our AAV-HLP-mGHR–treated GHR-/- mice, Igf1 expression was increased but reached only ~40–45% of wild-type liver levels, which may be insufficient to fully restore IGF1-mediated feedback inhibition of pituitary GH secretion. Under such conditions, pituitary GH output would be expected to increase to compensate for partial IGF1 deficiency, with the potential to override the normal male, high-amplitude, low-baseline GH pulse pattern, resulting in a more continuous GH profile that functionally resembles the female pattern. Loss of a robust male-pattern GH profile would be sufficient to disrupt male-biased hepatic gene expression even in the context of partially restored hepatocyte GHR expression. However, we were not able to test this hypothesis, as pituitary GH secretion profiles are technically challenging to measure in intact mice, requiring frequent serial blood sampling without introduction of stress.

Our findings of increased expression of cancer-associated genes in AAV-HLP-mGHR-treated livers compared to AAV-HLP-Luc-treated livers are surprising and novel. We acknowledge that, from a hepatic cancer viewpoint, the HLP promoter restricts oncogenic actions to the liver; however it remains important to examine the upregulation of oncogenes in the context of GH signaling, as the upregulated proto-oncogenes may contribute to the transformation ability of GH signaling in hepatocellular cancer. We observed significantly increased expression of 20 genes associated with cancer, covering a wide range of cancer types (**Table 1**), even though GHR levels were only 5% restored at the RNA level, and GHR protein levels were also very low following AAV-HLP-mGHR treatment. This is consistent with prior findings of increased GHR expression in a range of cancers, including malignant melanoma, B- and T-cell lymphoma, squamous cell lung carcinoma, and others (50). Oncomine data also indicate overexpression of GHR and GH in a variety of cancers, including prostate cancer, glioblastoma, T cell lymphoma, colon and ovarian cancers (51). These findings are consistent with observations that GHR function-deficient Laron dwarfs do not suffer from cancer and that rodents lacking functional GH or its receptor are less susceptible to cancers, including lymphoma, lung adenocarcinoma, mammary carcinoma and colon adenocarcinoma (51–54). Further study is needed to understand precisely why expression of GHR at a low level strongly induces certain cancer-associated genes, and whether cancer-associated gene induction might be reduced or avoided at higher, wild-type liver levels of GHR.

Importantly, AAV-HLP-mGHR treatment largely increased the expression of cancer-associated genes toward levels comparable to wild-type (GHR+/+) mice, whereas the AAV-HLP-Luc control vector did not induce oncogene upregulation relative to untreated GHR-/- Laron dwarf mice (**Supplementary Table 2**), supporting the safety profile of AAV vectors for gene therapy applications. However, AAV-HLP-mGHR increased expression of a small subset of genes beyond GHR+/+ levels (p-adj < 0.05), including proto-oncogenes such as Ascl1, Tmprss4 and others. Among these, Ascl1 was the only cancer-associated gene consistently elevated in both male and female mice compared with GHR+/+ controls, exhibiting a 4000-fold increase (log2FC: 11.97; p-adj: 5.69E-33) relative to wild-type GHR+/+ mice (mean FPKM in GHR+/+: 0 vs. mean FPKM in AAV-HLP-mGHR: 8.01). Ascl1 is highly expressed in SCLC, glial tumors and medullary thyroid cancer. In SCLC, Ascl1 drives the expression of protooncogenes including Ret, Mycl1, Sox2 and Bcl2, and is associated with treatment resistance (55); however, we did not observe upregulation of those genes in the AAV-HLP-mGHR-treated livers. While there are data suggesting that Ascl1 acts as a tumor suppressor in HCC (56), other evidence indicates that Hepatitis B virus promotes HCC progression through Ascl1 (57). The large fold change likely reflects negligible basal expression of Ascl1 in healthy, non-pregnant adult liver. Ascl1 is physiologically induced in the maternal liver to support a healthy pregnancy, showing marked upregulation (~26,000-fold on gestational days 13 and 15 relative to the prepregnancy state), where it plays a key role in regulating metabolic adaptations during pregnancy (58). Restoration of liver-specific mGHR expression via AAV-mediated gene therapy may therefore trigger a similar physiological transcriptional response that is not necessarily associated with increased hepatocellular carcinoma risk. Nevertheless, as elevated expression of Ascl1 has been associated with hepatitis B–related hepatocellular carcinoma (57), additional long-term studies are required to exclude potential oncogenic risks. It is highly relevant that hepatocellular cancer is known to be promoted by GH and use of pegvisomant is a therapeutic strategy for HCC treatment (59).

In summary, while there is improvement of body growth in GHR-deficient Laron mice after therapy with AAV-HLP-mGHR, whole transcriptome data showed unexpected differences in expression of growth and cancer-associated genes compared to metabolism-related genes, as well as significant differences in the impact of AAV-HLP-mGHR treatment on liver gene expression between male and female mice. Although some of these differences may be due to insufficient GHR RNA expression, the very high expression of some genes may be, in part, due to a constitutively active promoter without normal regulation of the endogenous GHR gene. Given the heterogeneity of mGHR expression seen on immunohistochemistry (**Supplementary Figure 3**), more detailed single-cell level analyses are an important area for future investigation, to determine optimum transfection levels and evaluate the diversity of expression of potential oncogenes and sex-related genes in individual cells. These findings may have implications for human gene therapies that routinely use such unregulated promoters.

## Data Availability

The datasets presented in this study can be found in online repositories. The names of the repository/repositories and accession number(s) can be found below: https://www.ncbi.nlm.nih.gov/geo/, GSE289769.

## Supplementary material

Supplementary Figure 1 Lack of difference in gene expression between AAV-HLP-Luc and GHR-/- mice. **(A)** Gene expression heatmap of 4 differentially expressed genes. **(B)** Gene set enrichment analysis of biological processes differentially enriched between AAV-HLP-Luc and GHR-/- mice.

Supplementary Figure 2 Rescue effect of a single AAV dose to the liver. **(A)** Gene expression heatmap of 240 on-target differentially expressed genes. **(B)** Gene set enrichment analysis of biological processes differentially enriched between AAV-HLP-Luc and GHR-/- mice, using only on-target genes.

Supplementary Figure 3 Immunohistochemistry analysis of mGHR expression in a representative liver section of GHR+/+, Laron mouse injected with AAV-HLP-mGHR, Laron mouse injected with AAV-HLP-Luc and GHR-/-. Livers were analyzed 25–26 weeks after AAV injection (corresponding to 30–31 weeks of age). For GHR+/+ and GHR-/-, livers were analyzed 16 weeks post-injection (corresponding to 21 weeks of age).

Supplementary Table 1 Genes rescued on-target.

Supplementary Table 2 Expression of cancer related genes.

Supplementary Table 3 Unaltered carbohydrate and lipid.

Supplementary Table 4 Expression of carbohydrate and lipid.

Supplementary Table 5 Distribution of sex-biased genes.
