# Supplementary figures and images for: GH-resistant (Laron) mice: gene therapy with a liver-specific GH receptor causes unbalanced upregulation of female-biased and growth-related genes
Source: Front Endocrinol (Lausanne). 2026 May 28;17:1808977. doi: 10.3389/fendo.2026.1808977 (PMC13253266; doi:10.3389/fendo.2026.1808977)

# Supplementary Figure 1

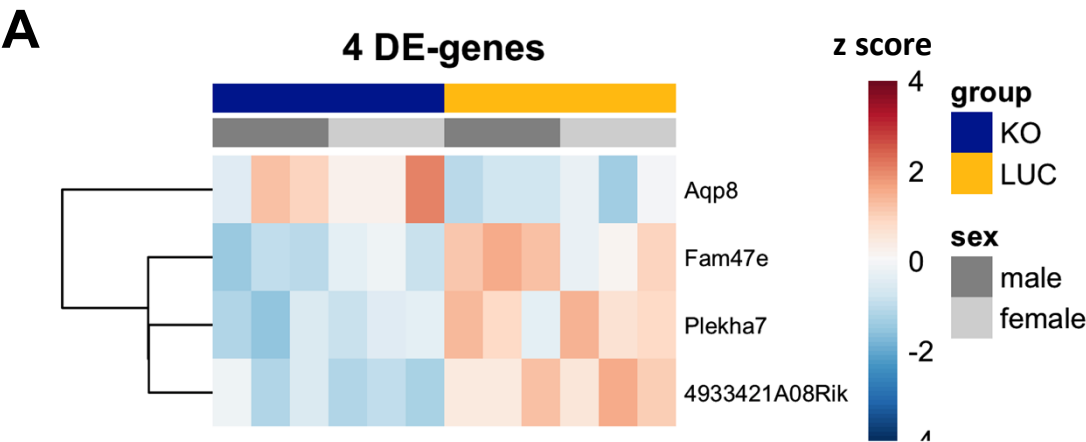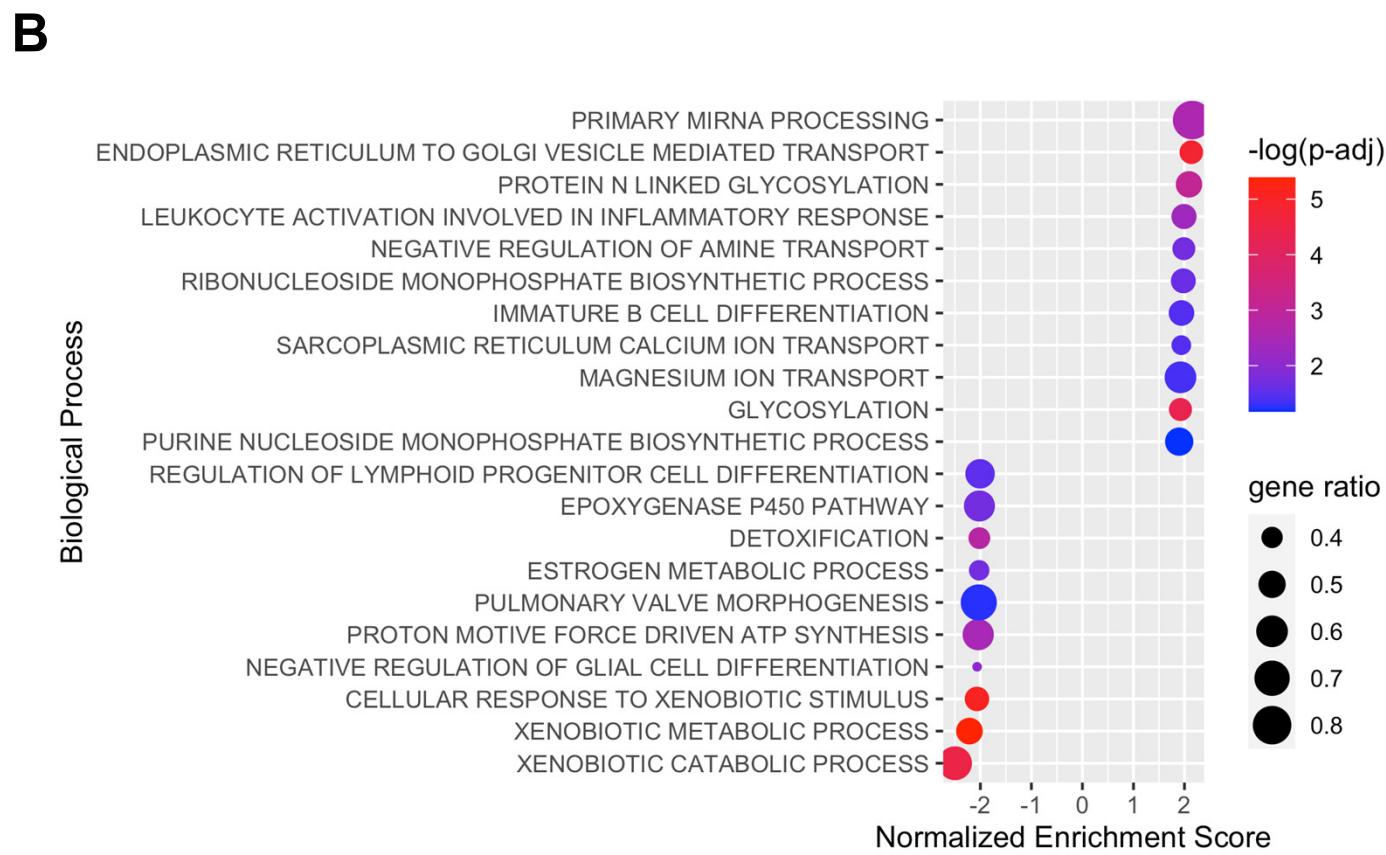

Supplement: Supplementary Figure 1 — Lack of difference in gene expression between AAV-HLP-Luc and GHR-/- mice. (A) Gene expression heatmap of 4 differentially expressed genes. (B) Gene set enrichment analysis of biological processes differentially enriched between AAV-HLP-Luc and GHR-/- mice. [file DataSheet1.pdf]

**A**

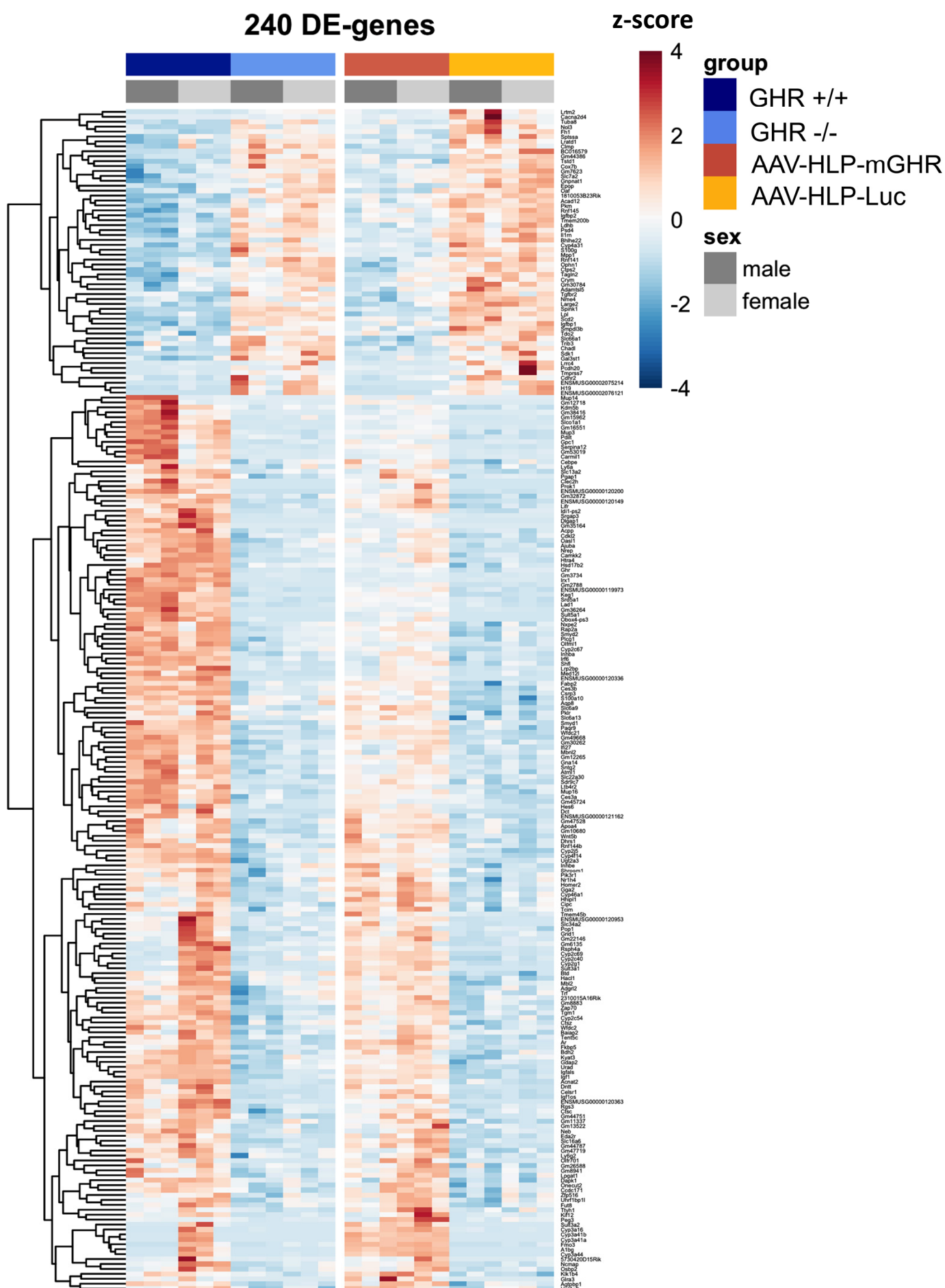

**B**

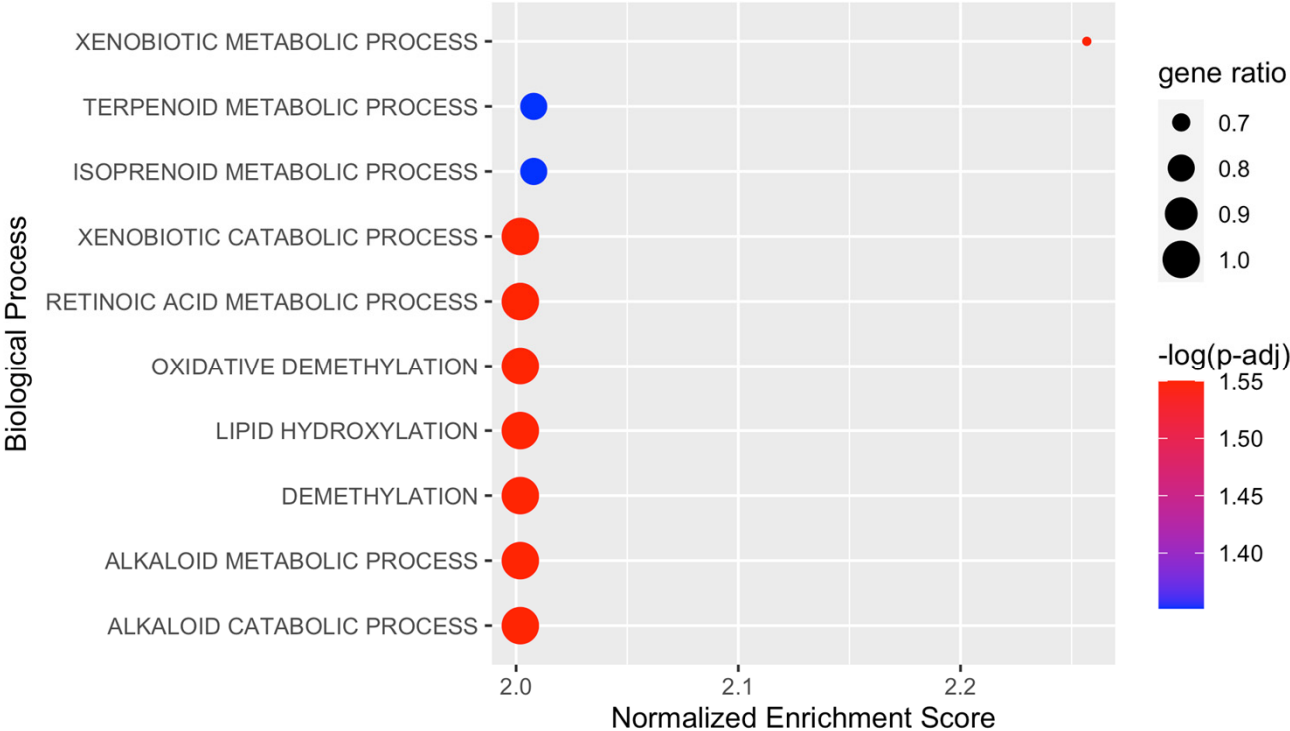

Supplement: Supplementary Figure 2 — Rescue effect of a single AAV dose to the liver. (A) Gene expression heatmap of 240 on-target differentially expressed genes. (B) Gene set enrichment analysis of biological processes differentially enriched between AAV-HLP-Luc and GHR-/- mice, using only on-target genes. [file DataSheet2.pdf]

## Supplementary Figure 3

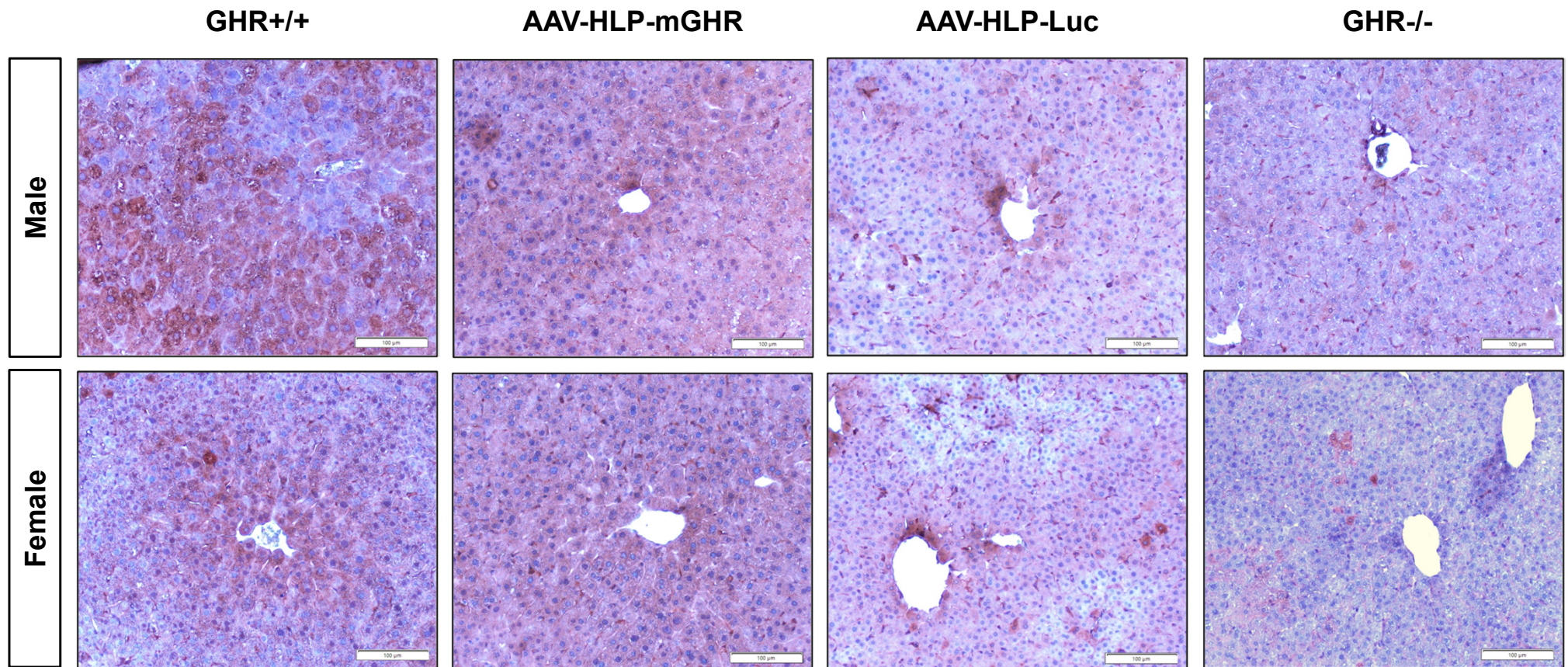

Supplement: Supplementary Figure 3 — Immunohistochemistry analysis of mGHR expression in a representative liver section of GHR+/+, Laron mouse injected with AAV-HLP-mGHR, Laron mouse injected with AAV-HLP-Luc and GHR-/-. Livers were analyzed 25–26 weeks after AAV injection (corresponding to 30–31 weeks of age). For GHR+/+ and GHR-/-, livers were analyzed 16 weeks post-injection (corresponding to 21 weeks of age). [file DataSheet3.pdf]
