# Supplementary material for: GH-resistant (Laron) mice: gene therapy with a liver-specific GH receptor causes unbalanced upregulation of female-biased and growth-related genes
Source: Front Endocrinol (Lausanne). 2026 May 28;17:1808977. doi: 10.3389/fendo.2026.1808977 (PMC13253266; doi:10.3389/fendo.2026.1808977)
Supplement: Supplementary Table 1 — Genes rescued on-target. [file DataSheet4.pdf]

Supplementary Table 1. Genes rescued on-target

Male & Female Mice									
		GHR+/+ vs GHR-/-		AAV-HLP-mGHR vs AAV-HLP-Luc		mean fpkm			
ENS ID	Gene	log2FC	p-adj	log2FC	p-adj	GHR+/+	GHR-/-	AAV-HLP-mGHR	AAV-HLP-Luc
ENSMUSG00000100277	1810053B23Rik	-3.626471128	0.000232462	-1.139370198	8.23096E-06	0.186990845	2.212769094	1.495011707	3.286768007
ENSMUSG00000117879	2310015A16Rik	1.604974395	0.028514297	1.45564813	0.00096493	15.20880188	4.978823958	14.31911967	5.171987528
ENSMUSG00000097766	5730420D15Rik	3.923370828	0.002270558	4.505452998	0.003538624	0.14995126	0.009786745	0.135175967	0.00526181
ENSMUSG00000022347	A1bg	13.29125126	9.95222E-06	14.55511917	2.94995E-62	173.2745326	0.0169753	138.7133271	0.005520957
ENSMUSG00000042647	Acad12	-0.816745981	5.21406E-06	-0.581503806	0.005886027	1.501517347	2.636395199	2.173330684	3.218407911
ENSMUSG00000060317	Acnat2	2.50605899	3.85126E-07	1.66766071	0.004064077	21.27061468	3.662623309	13.42763277	4.229533933
ENSMUSG00000032561	Acpp	2.689007216	3.47649E-10	1.257364813	0.002957211	2.006358403	0.312956565	0.851958142	0.349484741
ENSMUSG00000043822	Adamtsl5	-0.991619183	0.023951665	-0.983298307	0.048500314	0.114917867	0.226893796	0.149347557	0.290657917
ENSMUSG00000028184	Adgrl2	0.701993299	1.95596E-05	0.471703719	0.003436104	3.683462834	2.266847152	3.268282918	2.355081243
ENSMUSG00000021557	Agtpbp1	0.613796662	1.01677E-06	0.593201456	0.025509879	0.465033583	0.304277833	0.491077995	0.325100881
ENSMUSG00000022178	Ajuba	1.814946037	6.20981E-23	1.014568436	0.00098845	2.060616128	0.587785873	0.961950202	0.470874632
ENSMUSG00000032080	Apoa4	1.561130981	4.52241E-08	1.213860154	2.76403E-06	112.03531	38.00414216	107.216472	45.58824961
ENSMUSG00000030762	Aqp8	1.020606684	0.036752163	1.432817583	4.85234E-13	91.27850526	44.32190098	51.40625717	19.05375076
ENSMUSG00000046532	Ar	3.835417448	4.28022E-07	3.143555471	5.15966E-08	1.502527443	0.106566814	1.921949735	0.222477413
ENSMUSG00000054843	Atrnl1	0.907931022	1.35947E-06	0.41072609	0.018435677	5.552596567	2.975014444	4.008758074	2.992457407
ENSMUSG00000025372	Baiap2	1.116257574	0.000167777	0.627566761	0.012183426	3.615868416	1.656269375	3.01125236	1.926414141
ENSMUSG00000033187	BC016579	-4.381232415	6.33586E-07	-3.111376367	8.25905E-06	0.008824827	0.203973735	0.030189132	0.266481012
ENSMUSG00000028167	Bdh2	1.101369016	2.03959E-13	0.734982305	0.038047289	3.203207694	1.488110128	2.568184732	1.559054212
ENSMUSG00000025128	Bhlhe22	-4.081835759	0.000104158	-2.583532932	8.23096E-06	0.01101447	0.18719798	0.06709097	0.405537007
ENSMUSG00000021900	Btd	0.432847437	0.012641551	0.589855255	0.002850417	30.82973859	22.63167902	27.00697157	17.96017138
ENSMUSG00000041460	Cacna2d4	-3.968928611	0.003093876	-3.45737557	0.006170955	0.000734854	0.015970597	0.010231688	0.10808965
ENSMUSG00000029471	Camkk2	1.572081013	7.3437E-25	0.923799879	0.00266872	1.091063197	0.366284535	0.65710805	0.34436003
ENSMUSG00000021338	Carmil1	3.794300375	1.15572E-12	1.161381348	0.018895133	1.364542114	0.101029027	0.2166713	0.09562241
ENSMUSG00000052407	Ccdc171	1.125956864	0.000241364	1.047861202	0.018039172	0.744829246	0.341695289	0.769899987	0.376093662
ENSMUSG00000034918	Cdhr2	-2.244906854	0.001390503	-1.666620876	0.041342626	0.066571324	0.31166092	0.104305154	0.321757569
ENSMUSG00000029403	Cdkl2	0.860048478	2.68565E-06	0.548274145	0.005886027	1.316270707	0.723307918	0.938952043	0.639594092
ENSMUSG00000052435	Cebpe	1.038678562	0.002911405	0.973953256	0.020404573	4.822025956	2.404190114	3.924069575	2.036532651
ENSMUSG00000016028	Celsr1	3.93972853	3.17521E-40	3.599829481	1.9855E-27	1.328654447	0.085971479	0.946595889	0.077831112
ENSMUSG00000069922	Ces3a	3.334595154	3.17834E-09	1.510722019	8.32531E-05	505.9338047	50.53566215	137.0868819	47.8467328
ENSMUSG00000062181	Ces3b	3.973247289	2.01718E-09	2.718278119	0.010850258	135.1078494	8.894423997	25.30134651	3.887438449
ENSMUSG00000063765	Chadl	-1.02521386	0.000392953	-1.001804713	0.000378979	0.144755127	0.292262261	0.146867175	0.292477298
ENSMUSG00000034157	Cipc	0.704244914	7.96993E-06	0.576642264	0.003495883	6.020063432	3.698560393	6.012443163	4.040063561
ENSMUSG00000030364	Clec2h	7.980511226	2.97686E-15	8.211673644	2.54363E-16	3.197464396	0.012875697	1.107893149	0.003735263
ENSMUSG00000032024	Clmp	-1.012827883	0.007482051	-0.829636815	0.018084325	0.275010764	0.548283872	0.266668904	0.471988352
ENSMUSG00000031231	Cox7b	-0.602605905	0.000366101	-0.363549167	0.033031469	78.12119154	117.1472608	87.54990833	112.4535532
ENSMUSG00000030905	Crym	-1.436484259	0.000991434	-0.93119474	0.042119354	0.220690989	0.592818793	0.414769034	0.777743511
ENSMUSG00000030470	Csrp3	2.835035068	4.27635E-09	2.769526806	0.000802486	5.830401524	0.812420533	2.347820174	0.35055913

ENSMUSG00000031360	Ctps2	-0.598448469	0.005341908	-0.677656233	0.001042158	2.342244696	3.504392077	2.283592564	3.606812755
ENSMUSG00000030560	Ctsc	1.570511749	5.30826E-09	0.919716684	2.81251E-05	26.14411617	8.835753235	19.96898154	10.52632983
ENSMUSG00000016256	Ctsz	0.333927181	0.010495405	0.375299465	0.001388557	144.2042121	114.1902408	135.0923216	104.0968103
ENSMUSG00000025004	Cyp2c40	3.60653343	0.030892374	4.859957246	2.97328E-09	96.249161	7.811043904	37.54117148	1.344217541
ENSMUSG00000067225	Cyp2c54	2.94280026	1.94675E-09	1.412341168	0.012327992	142.144213	18.63525613	66.24105191	24.77261628
ENSMUSG00000062624	Cyp2c67	1.301846139	2.87387E-11	0.541407214	0.011557782	145.7383086	60.04073074	75.90408693	52.39814111
ENSMUSG00000092008	Cyp2c69	6.748224708	0.00099618	7.951307814	1.76456E-15	292.7811007	2.705923538	75.37802112	0.319309364
ENSMUSG00000049685	Cyp2g1	4.32714301	3.15046E-05	5.318719478	8.54924E-34	3.533852249	0.170689409	2.944920651	0.072419544
ENSMUSG00000052520	Cyp2j5	0.549510325	7.85225E-06	0.632071956	0.00059211	262.2081837	179.250353	237.8621691	152.9751341
ENSMUSG00000038656	Cyp3a16	6.508708561	0.009990716	6.150340113	3.16017E-16	22.76979142	0.235876747	22.83259396	0.310595523
ENSMUSG00000075551	Cyp3a41a	11.88881222	0.001367262	13.36584801	3.40563E-50	26.23921459	0.006897473	26.16010833	0
ENSMUSG00000075552	Cyp3a41b	9.566671443	0.001322526	12.68677477	1.11208E-41	14.3187764	0.018023243	22.65200961	0.002206274
ENSMUSG00000054417	Cyp3a44	7.136241553	0.004505563	9.74176377	1.90834E-48	15.30315092	0.107255463	29.16957365	0.033021812
ENSMUSG00000021259	Cyp46a1	1.516972114	0.000393156	1.791576045	0.005975861	0.530404573	0.182502452	0.809442942	0.237707538
ENSMUSG00000028712	Cyp4a31	-2.577928541	8.38422E-05	-1.917586856	0.011169086	2.423769369	13.9474349	4.84999662	18.08499777
ENSMUSG00000024292	Cyp4f14	2.608620976	1.92827E-27	1.549499582	1.5483E-12	123.1832235	20.22253874	52.89457549	17.93749657
ENSMUSG00000021559	Dapk1	0.601652734	2.71842E-05	0.606412194	0.025563034	7.881294391	5.163736103	6.559151274	4.282708431
ENSMUSG00000022129	Dct	10.2890877	2.61241E-23	9.299980526	2.29221E-20	1.057740818	0	0.540247362	0
ENSMUSG00000002332	Dhrs1	0.582129036	0.000228121	0.581626357	0.0003229	46.81791989	31.29943755	43.55881884	29.17101125
ENSMUSG00000003279	Dlgap1	9.683533727	7.92283E-27	7.029152219	5.02937E-12	0.229709358	0	0.037704069	0
ENSMUSG00000025014	Dntt	5.295783713	1.83147E-09	5.033392474	3.46448E-15	1.757327024	0.043941516	0.857884377	0.026380904
ENSMUSG00000034457	Eda2r	2.278016267	2.34368E-09	2.10605645	0.001124086	0.234544411	0.048700928	0.195086465	0.045339799
ENSMUSG00000119973	ENSMUSG0000011	3.525318977	7.45382E-23	2.026025803	6.0126E-05	1.821638807	0.159261472	0.626936734	0.152175696
ENSMUSG00000120149	ENSMUSG0000012	4.415645295	1.02262E-31	4.296350483	4.16802E-17	3.625326129	0.16642651	2.79944718	0.139072405
ENSMUSG00000120200	ENSMUSG0000012	11.39485156	1.44344E-22	5.536670082	0.000372901	6.765674383	0	1.997346043	0.040634412
ENSMUSG00000120336	ENSMUSG0000012	5.900056973	2.8556E-09	4.679965283	0.00074587	0.636960869	0.006133094	0.200064033	0
ENSMUSG00000120363	ENSMUSG0000012	6.794044208	1.25963E-10	5.854099817	2.25469E-07	1.029161149	0.006051004	0.387044419	0
ENSMUSG00000120953	ENSMUSG0000012	3.751841058	1.46381E-06	4.575757745	6.4493E-06	0.756083479	0.053989235	0.365396098	0.014877686
ENSMUSG00000121162	ENSMUSG0000012	2.099877047	0.001427105	2.38486185	0.009072095	0.29057903	0.0667633	0.23122664	0.043460199
ENSMUSG00002075214	ENSMUSG0000207	-5.737532833	0.001046693	-5.413353048	0.00032039	0.049749638	5.139391754	0	2.876965654
ENSMUSG00002076121	ENSMUSG0000207	-6.504643925	0.000359616	-4.445217324	0.001225768	0	6.261974362	0.215517155	5.004366306
ENSMUSG00000043439	Epop	-0.694728346	0.002271155	-0.865086211	4.38552E-05	2.126670525	3.444553093	2.60133508	4.734318584
ENSMUSG00000023057	Fabp2	1.302549762	9.23017E-09	1.226863341	5.72542E-05	43.1024052	17.31726271	29.67123324	12.8718561
ENSMUSG00000026526	Fh1	-0.531515353	0.001528605	-0.861367306	0.022538424	48.27233731	69.89206746	55.72773953	99.95412468
ENSMUSG00000024222	Fkbp5	1.979289938	2.0088E-05	1.952909869	3.1767E-28	4.709737692	1.179109752	4.861748353	1.253234243
ENSMUSG00000026691	Fmo3	5.822652238	0.005859284	8.196714322	1.48899E-15	43.76544216	0.763765092	115.6418595	0.412191661
ENSMUSG00000021065	Fut8	0.762599958	0.005106271	0.967632538	0.000289823	0.616861416	0.362380879	0.602712722	0.309286106
ENSMUSG00000049721	Gal3st1	-1.656502439	5.8469E-05	-0.764936204	0.04907594	0.560549664	1.741589784	0.77108164	1.30903872
ENSMUSG00000027865	Gdap2	0.729130939	1.48957E-16	0.460386911	0.001862442	5.862906158	3.532899397	4.522568778	3.28462799
ENSMUSG00000030872	Gga2	0.611190538	0.01864545	0.97159009	1.83789E-12	1.887237522	1.233522499	2.157137126	1.098879346
ENSMUSG00000055737	Ghr	3.224848497	7.3516E-136	0.659434724	0.009606139	65.8444543	7.023329947	11.65728154	7.423425348
ENSMUSG00000038257	Glra3	3.788611617	0.031855252	5.137839983	0.00072221	0.007425718	0	0.019260688	0
ENSMUSG00000074373	Gm10680	1.5202851	8.70788E-05	1.112881127	0.000672806	26.27215429	9.158415796	23.24268029	10.60362332

ENSMUSG00000087173	Gm11337	1.144327473	0.027828811	1.79320276	0.001145781	1.959576202	0.900242446	1.80742134	0.514886536
ENSMUSG00000085132	Gm12265	2.822607984	3.97604E-14	1.890854644	3.28949E-06	1.815658594	0.258292525	0.92758689	0.247445748
ENSMUSG00000086136	Gm12718	5.870824523	0.001901291	5.251843982	0.000756027	2.480907318	0.044409405	0.370009969	0.00751324
ENSMUSG00000086447	Gm13522	2.107627796	7.76802E-08	1.501780702	0.029638631	1.42947826	0.334060163	1.301604025	0.449275876
ENSMUSG00000084981	Gm15962	3.773469004	0.006126166	3.711917979	0.00444009	1.165074795	0.091606473	0.206444148	0.015025579
ENSMUSG00000066477	Gm16551	6.190560248	5.82811E-14	2.429577808	0.000289823	1.6688459	0.024029087	0.215754985	0.038765395
ENSMUSG00000117084	Gm22146	3.437755791	0.003413675	3.528215351	7.88101E-14	1.874966994	0.172775787	1.752477327	0.151298214
ENSMUSG00000097730	Gm26588	0.613159064	0.030842427	1.07210273	0.032136021	1.373511415	0.889671846	1.618607639	0.759619803
ENSMUSG00000085995	Gm2788	4.834222471	1.0779E-16	3.905604279	0.005950981	1.635905575	0.05791694	0.331653944	0.02263159
ENSMUSG00000112249	Gm30262	2.41637775	7.51685E-09	1.114360664	0.004640221	5.785516475	1.122773103	3.119736173	1.407831291
ENSMUSG00000107516	Gm30784	-0.590853586	0.000992498	-0.683639116	0.044950603	2.33005075	3.505755547	2.794949768	4.431007495
ENSMUSG00000112793	Gm32872	2.385429023	1.44736E-07	2.837790116	8.88641E-06	5.728163426	1.085963616	3.561654726	0.481009498
ENSMUSG00000114818	Gm35164	7.942223798	8.28741E-16	4.088147943	0.037991743	0.395332694	0	0.02826176	0
ENSMUSG00000113543	Gm36264	4.228153599	1.23265E-27	1.818386255	0.000263096	1.02655154	0.055352902	0.191267757	0.054274357
ENSMUSG00000117780	Gm3734	5.773685821	1.66783E-37	1.572245759	0.00713566	6.323242509	0.116076683	0.353108868	0.118672718
ENSMUSG00000110151	Gm38416	6.8018988	6.29421E-09	3.271787792	0.037878722	0.506852652	0.004788446	0.076964125	0.007585353
ENSMUSG00000107689	Gm44386	-3.030780758	0.003462544	-2.854005588	0.010521569	0.040123922	0.333333285	0.054102874	0.356554269
ENSMUSG00000109244	Gm44751	1.750474706	8.94701E-06	1.185254624	0.002946692	0.283399591	0.082738053	0.239794166	0.105551443
ENSMUSG00000108822	Gm44787	3.605716869	3.03934E-07	4.334706303	7.41467E-19	2.772678429	0.226250148	2.854962673	0.140185487
ENSMUSG00000110488	Gm45724	3.257506452	7.76802E-08	1.318860478	0.042772383	12.67478144	1.340550717	2.977332332	1.197811522
ENSMUSG00000111282	Gm47528	1.507713275	0.00013422	1.19468454	0.002454896	1.449936321	0.513569503	1.272319795	0.552972382
ENSMUSG00000112599	Gm47719	1.361451566	0.000212502	1.550873869	0.033031469	0.681521651	0.263515041	0.485155402	0.163831346
ENSMUSG00000116718	Gm49668	3.127173381	1.61701E-09	2.950148316	1.15405E-25	2.643439783	0.3053057	1.505976668	0.195029145
ENSMUSG00000118631	Gm53019	6.991708408	2.03959E-13	2.892871266	0.000109705	6.481023401	0.053084081	0.491726335	0.064253927
ENSMUSG00000106069	Gm6135	4.153541432	0.000698377	2.729656143	0.001963219	22.68412207	1.22666478	5.399943962	0.834416683
ENSMUSG00000118283	Gm7623	-0.391802377	0.02837872	-0.448320369	0.006646078	5.567397792	7.253470704	6.516528817	8.877128855
ENSMUSG00000099707	Gm8883	1.361197746	3.50997E-09	1.196327742	6.52428E-06	8.003856291	3.135140738	6.899148421	2.969502798
ENSMUSG00000097426	Gm8941	2.238403483	0.000157857	1.683254042	0.000225545	5.687403094	1.22953029	4.815888485	1.481008157
ENSMUSG00000024697	Gna14	2.229679099	4.19538E-10	1.07215917	0.004508387	1.549836183	0.335749104	0.730391057	0.346415567
ENSMUSG00000037722	Gnpnat1	-0.405448678	0.000204518	-0.346985335	0.040146496	14.50427731	19.12863469	16.71997744	21.18992344
ENSMUSG00000034220	Gpc1	3.378810841	1.02899E-09	0.966682407	0.04907594	2.012122971	0.202079024	0.485278709	0.25014981
ENSMUSG00000041078	Grid1	2.978960101	0.001948262	2.327654116	8.73046E-08	0.528223427	0.066240892	0.52217774	0.104183822
ENSMUSG00000000031	H19	-6.090529558	1.51648E-05	-5.615939741	1.84618E-09	0.153479322	9.92499202	0.178146395	8.698027302
ENSMUSG00000021884	Hacl1	0.903051105	0.001609679	1.439840659	2.26005E-08	5.059179086	2.671614115	4.553896461	1.687032351
ENSMUSG00000067071	Hes6	1.392740899	0.000334641	0.637481137	0.010819752	38.6990905	14.81280784	23.53081664	15.08022605
ENSMUSG00000021260	Hhipl1	1.164642373	0.016469772	1.978875983	7.69719E-05	0.271016215	0.121199707	0.440420018	0.112220333
ENSMUSG00000025813	Homer2	1.442744141	0.000418166	1.61754882	4.41472E-08	3.565005796	1.314014973	3.545458729	1.160446903
ENSMUSG00000031844	Hsd17b2	1.653768984	8.22083E-11	0.716768967	0.033930849	86.8689738	27.8119777	31.28698942	19.20935019
ENSMUSG00000037406	Htra4	3.746557734	5.26775E-12	3.083693698	0.00166767	0.493180564	0.036030236	0.219614213	0.024981249
ENSMUSG00000104664	Idi1-ps2	3.801340701	1.31881E-10	2.628097497	0.001546655	1.480413206	0.107493574	0.616571759	0.098785094
ENSMUSG00000064215	Ifi27	1.661116244	4.91181E-23	0.642423636	0.039748008	57.00610029	18.15751724	29.47548175	18.56334322
ENSMUSG00000020053	Igf1	4.652319435	2.73457E-18	4.028675136	3.17985E-16	64.3566781	2.639043213	26.73390147	1.662986209
ENSMUSG00000058934	Igf1os	3.373830035	2.22261E-11	4.310108548	2.90187E-09	0.302341841	0.029495781	0.274177905	0.013033442

ENSMUSG00000046070	Igfals	4.579516865	1.09454E-23	3.968927585	2.49112E-18	45.8902325	1.947673301	20.64121553	1.351211711
ENSMUSG00000020429	Igfbp1	-3.361974718	7.41986E-08	-2.241532083	0.004852789	15.49989088	153.3165109	77.83042185	367.7602839
ENSMUSG00000039323	Igfbp2	-1.73055495	5.48973E-10	-1.212206951	6.37639E-07	181.322949	592.8141512	312.3313695	724.9572369
ENSMUSG00000026981	Il1rn	-2.185992493	5.92818E-07	-1.128607667	0.003895868	0.561029567	2.510341553	1.505683428	3.275024969
ENSMUSG00000041324	Inhba	2.603698555	5.63121E-22	1.508932565	0.000376023	2.040933479	0.34083474	1.041820021	0.364189551
ENSMUSG00000047492	Inhbe	0.726879124	0.015239225	1.385595923	8.0833E-05	9.04934643	5.575695707	9.572481752	3.717690818
ENSMUSG00000026638	Irf6	1.870112374	2.22979E-29	0.993719711	0.019511229	6.016723135	1.654295847	2.554870945	1.276659308
ENSMUSG00000060969	Irx1	8.202549752	4.50742E-19	3.012917307	0.026840695	0.78002	0.001807735	0.056160794	0.006659313
ENSMUSG00000042207	Kdm5b	0.865307766	3.94035E-05	0.381787712	0.012418753	1.381587938	0.765323297	0.802644907	0.613624979
ENSMUSG00000024694	Keg1	4.49587539	4.67346E-18	2.328236255	4.80121E-34	27.7661335	1.276503466	5.1470034	1.032206674
ENSMUSG00000028357	Kif12	3.067393015	0.000141233	2.213127512	0.028371102	0.089202311	0.010877017	0.114021978	0.023836421
ENSMUSG00000066513	Klk1b4	0.654045244	0.012534643	1.431055233	1.94459E-05	3.379489444	2.131893733	4.523773335	1.669529383
ENSMUSG00000040213	Kyat3	1.51512454	3.59648E-08	1.111004407	0.002873503	19.2101364	6.797925443	12.089973	5.732064569
ENSMUSG00000041782	Lad1	4.858558099	5.30679E-11	2.203645694	2.25075E-05	0.975234074	0.034412825	0.269619868	0.058196374
ENSMUSG00000040434	Large2	-0.999512524	0.011217631	-1.455520423	0.000289823	0.08420624	0.165905927	0.098391125	0.267558167
ENSMUSG00000030246	Ldhb	-1.728167625	3.49544E-08	-1.529336741	9.10709E-07	1.635008554	5.350729266	2.284189005	6.584059214
ENSMUSG00000054263	Lifr	1.526974765	3.02111E-12	1.604119933	1.96747E-14	33.83067566	11.76688147	32.9148817	10.77714336
ENSMUSG00000026623	Lpgat1	0.567441261	0.006422826	0.56632462	0.000790468	33.45199051	22.64482454	36.05962944	24.23863711
ENSMUSG00000015568	Lpl	-2.584953933	2.38919E-10	-2.586961024	9.08052E-17	2.355522488	13.80496203	2.685733661	15.7337342
ENSMUSG00000020607	Lratd1	-1.324977858	0.004738807	-1.062582273	0.001949298	0.397195724	0.968887513	0.535800245	1.105512346
ENSMUSG00000031637	Lrp2bp	3.466377166	1.139E-14	2.290949922	0.010443783	0.62230849	0.055380296	0.185947774	0.038308944
ENSMUSG00000049939	Lrrc4	-3.246344249	0.010646252	-5.441266823	6.76395E-06	0.006691591	0.069176246	0.003333586	0.139781944
ENSMUSG00000055003	Lrtm2	-3.958507389	0.000104158	-3.599077665	0.000433642	0.045655809	0.704375256	0.287706087	3.30148013
ENSMUSG00000040432	Ltb4r2	1.457451173	1.19459E-08	0.862401962	0.04998213	0.453204988	0.163792836	0.301965635	0.166101594
ENSMUSG00000075602	Ly6a	1.505302266	0.031485655	0.775750125	0.014594048	15.98900665	5.843359811	9.128717926	5.32852124
ENSMUSG00000047728	Ly6g2	0.712951367	0.002047993	0.552311259	0.019884899	63.85134248	38.70885525	53.04071722	35.78943044
ENSMUSG00000024863	Mbl2	0.778303644	0.037690957	0.954890232	1.18703E-06	453.6453333	260.1532594	347.7587077	181.0365614
ENSMUSG00000022139	Mbnl2	1.291487518	3.78257E-14	0.627506501	0.022028585	3.345638539	1.379612726	2.395180341	1.536115257
ENSMUSG00000056476	Med12l	4.248761396	2.87718E-33	1.93362747	0.01420586	0.26385807	0.013810661	0.10475984	0.027482569
ENSMUSG00000031402	Mpp1	-0.59062811	4.01961E-06	-0.512188411	0.001050332	5.76836109	8.614480529	6.856246345	9.739206073
ENSMUSG00000073830	Mup14	12.11628852	8.6308E-12	6.441748795	0.001809708	324.6621179	0.0743646	2.34242885	0.026450335
ENSMUSG00000078675	Mup16	5.649355866	8.59798E-12	2.73550291	0.023887138	449.3890787	8.921154738	51.20089288	7.527264143
ENSMUSG00000066154	Mup3	4.436627961	1.24462E-21	1.521222273	7.67558E-08	5693.634231	267.588932	604.2799573	212.0131089
ENSMUSG00000043924	Ncmap	2.386152592	0.000916737	2.45117701	0.000239639	0.491905595	0.093388506	0.366370673	0.06544896
ENSMUSG00000026950	Neb	1.802012427	0.000405008	1.52195545	0.033714963	0.539775908	0.157681563	0.395385605	0.137048157
ENSMUSG00000024177	Nme4	-0.818975127	0.001915258	-1.032830431	0.000103981	0.788512128	1.391618214	0.90648507	1.839073059
ENSMUSG00000014776	Nol3	-1.183689454	0.00048269	-1.150754548	0.001323088	0.261335185	0.58906551	0.427545011	0.933671458
ENSMUSG00000047638	Nr1h4	0.357345855	0.011440221	0.46365604	0.039214709	43.89834752	34.35034211	44.02639324	32.01570869
ENSMUSG00000042834	Nrep	3.719211156	7.78254E-14	1.865288358	0.009565344	7.039929642	0.543129939	1.825570981	0.493554322
ENSMUSG00000032028	Nxpe2	1.584163719	3.2514E-10	0.913880604	0.044021476	6.52666168	2.198953622	3.285487297	1.762645781
ENSMUSG00000032014	Oaf	-0.284367271	0.033792719	-0.292094058	0.04907594	85.1930889	103.6364328	85.85922275	104.9641824
ENSMUSG00000041827	Oasl1	1.763738688	3.28672E-09	0.990214052	0.00025737	5.087490519	1.515326967	2.637302766	1.310923584
ENSMUSG00000099242	Obox4-ps3	5.577917961	4.38643E-08	4.047898443	0.03455317	0.887749974	0.017803915	0.163893001	0

ENSMUSG00000051041	Olfml1	2.176585116	1.38946E-18	0.516497266	0.02724007	10.70621698	2.399492866	3.108189397	2.165275902
ENSMUSG00000036744	Olfr701	5.595368875	2.85754E-05	5.71100376	3.59146E-05	0.056597289	0	0.064058358	0
ENSMUSG00000045991	Onecut2	0.703746546	1.1179E-05	0.658705297	0.023361809	5.852897195	3.595844069	5.483788766	3.478051789
ENSMUSG00000031214	Ophn1	-0.601066478	0.002394183	-0.585175695	0.030058683	0.126536392	0.191835691	0.132524081	0.198064665
ENSMUSG00000020435	Osbp2	1.526583841	0.002450953	1.453154119	0.000513495	0.328714133	0.113375967	0.295812031	0.107310319
ENSMUSG00000064225	Paqr9	2.403615734	1.27155E-14	1.904860073	3.67888E-07	40.99890508	7.690068743	23.55388644	6.225162955
ENSMUSG00000050505	Pcdh20	-3.967892683	0.00024077	-2.703885483	0.001185027	0.004618731	0.07482789	0.02336169	0.155431515
ENSMUSG00000030968	Pdilt	1.902695926	2.437E-09	1.002757683	0.001963219	2.46884744	0.663622398	0.926742964	0.461707176
ENSMUSG00000002265	Peg3	0.635772737	0.02419149	1.924083648	0.04926639	0.157985319	0.101345918	0.509032886	0.132711949
ENSMUSG00000073678	Pgap1	0.969317132	2.2031E-06	0.653736815	0.026790345	1.552102795	0.788250909	1.338950257	0.855245257
ENSMUSG00000041417	Pik3r1	0.571721407	0.003973558	0.698289752	0.000889282	9.478124944	6.395353327	9.857171731	6.115957128
ENSMUSG00000041237	Pklr	1.023087751	0.000459371	0.97839183	0.003299945	98.86719008	48.11841041	67.48219626	34.31530956
ENSMUSG00000032294	Pkm	-0.682090011	5.88115E-06	-0.484452172	0.011405479	3.836094668	6.137439303	5.072039059	7.049964054
ENSMUSG00000016933	Plcg1	1.026463702	1.0641E-12	0.507004149	0.00017187	6.098082647	3.012601212	4.265993308	3.000662098
ENSMUSG00000022325	Pop1	0.687191993	0.006373416	0.589944763	0.041342626	1.116454796	0.686426134	1.013446667	0.671718212
ENSMUSG00000070368	Prok1	10.03699037	4.32839E-26	8.316010697	2.55242E-08	1.589731068	0	0.730369359	0.001263635
ENSMUSG00000026979	Psd4	-1.084733726	1.44911E-07	-0.550611153	0.017286688	0.327502947	0.686315468	0.486632969	0.712198777
ENSMUSG00000051615	Rap2a	0.924090755	3.4567E-07	0.469608829	0.005278876	6.825663014	3.612995344	4.746537889	3.449766763
ENSMUSG00000059810	Rgs3	1.550504506	1.97296E-09	0.761619877	0.000513495	1.220389565	0.414246606	0.701234945	0.412422885
ENSMUSG00000030788	Rnf141	-0.309525383	2.20624E-05	-0.22242429	0.030769714	5.39095246	6.669651803	5.690309257	6.623859467
ENSMUSG00000038068	Rnf144b	0.784515298	3.63962E-05	0.479244762	0.040146496	7.455958685	4.340810796	6.476989374	4.629975358
ENSMUSG00000019189	Rnf145	-1.104523394	8.43453E-06	-0.597736155	0.038422894	3.896710974	8.197347225	6.277755475	9.534933035
ENSMUSG00000039552	Rsph4a	2.875404469	0.018221767	3.574356455	3.26213E-10	0.280682153	0.035791639	0.222539393	0.018538466
ENSMUSG00000041959	S100a10	0.790683706	0.000335334	1.042626494	0.000626731	45.87214356	26.55207923	32.01006906	15.55174528
ENSMUSG00000040808	S100g	-5.089658243	2.73498E-06	-1.962593917	2.55242E-08	0.070167246	2.454030198	0.529533292	2.065059543
ENSMUSG00000025203	Scd2	-3.741661515	2.29093E-18	-2.492734674	0.017161921	2.092729473	27.02457144	8.726671109	47.96812627
ENSMUSG00000039683	Sdk1	-3.733250626	2.39806E-13	-2.584210708	3.22298E-06	0.013354677	0.181486731	0.024364988	0.148887028
ENSMUSG00000040127	Sdr9c7	2.451729102	2.68501E-09	1.40144987	0.025509879	19.44267569	3.635603588	8.684130511	3.307696717
ENSMUSG00000041567	Serpina12	5.721171053	2.05348E-11	3.271388198	7.26848E-21	16.31517253	0.316900277	2.289689035	0.234636222
ENSMUSG00000038884	Shfl	1.961397569	8.32349E-29	0.920609063	0.04907594	5.154193065	1.322361883	2.443334243	1.294289522
ENSMUSG00000018387	Shroom1	0.696254763	4.69704E-06	0.514359643	0.004857165	6.04061797	3.724987493	5.370149589	3.751271678
ENSMUSG00000001095	Slc13a2	1.794467918	3.87782E-08	1.849433058	0.002683029	0.770753666	0.223785352	0.413071216	0.115396453
ENSMUSG00000041920	Slc16a6	2.226327669	1.25896E-14	1.843983195	9.62642E-11	0.75894632	0.164101648	0.877852187	0.241566444
ENSMUSG00000052562	Slc22a30	1.328601358	2.1011E-13	0.602341806	5.09666E-05	33.03179615	13.19952655	21.19375563	14.00005586
ENSMUSG00000029188	Slc34a2	3.252823368	3.87025E-05	5.104370878	2.10583E-07	0.166468768	0.01738451	0.124580021	0.003608835
ENSMUSG00000028744	Slc66a1	-0.425813788	0.010395132	-0.484359264	0.012349461	2.8662506	3.817329122	2.640339426	3.684726149
ENSMUSG00000030108	Slc6a13	0.390791942	0.009400367	0.513061913	0.047899219	68.44206723	51.65190213	58.84759008	41.49051846
ENSMUSG00000028542	Slc6a9	1.372801352	9.5346E-07	1.792146261	1.5459E-12	8.100370655	3.082326603	7.44792591	2.181864427
ENSMUSG00000031596	Slc7a2	-1.106760471	9.90749E-07	-0.682879592	0.000181406	75.16463675	160.2031595	105.5722852	168.5670484
ENSMUSG00000041698	Slco1a1	12.13320772	3.46922E-35	5.814379141	1.55151E-24	66.35827843	0.015001112	0.96430911	0.01701107
ENSMUSG00000028885	Smpdl3b	-1.676523576	4.8021E-05	-1.547033377	0.000194945	0.214458281	0.683026777	0.416202056	1.201528395
ENSMUSG00000055027	Smyd1	2.765626107	5.24813E-15	1.039784655	0.04779276	1.030857504	0.152135655	0.522994647	0.250736153
ENSMUSG00000026603	Smyd2	1.10108462	2.28536E-19	0.717865805	0.00159097	4.884295994	2.279706052	3.04177352	1.852618864

ENSMUSG00000020672	Sntg2	3.311069338	4.97698E-18	1.386766019	0.001188164	1.938175764	0.200459667	0.791908511	0.298636752
ENSMUSG00000024503	Spink1	-7.928654038	4.5508E-33	-4.009077027	1.07634E-09	0.041196621	8.829187617	0.68713053	10.96208669
ENSMUSG00000044408	Sptssa	-0.377341249	0.007935561	-0.535749288	0.0098677	26.54359879	34.05031378	27.20050112	39.19883788
ENSMUSG00000021594	Srd5a1	3.215463722	2.07812E-19	0.973557021	3.48619E-06	19.03878175	2.073251368	4.211937613	2.131685776
ENSMUSG00000030257	Srgap3	3.890459507	1.15193E-34	1.304718453	0.020275672	0.657250335	0.043939964	0.149181891	0.060197299
ENSMUSG00000069668	Sult3a1	9.421825024	7.16913E-05	9.542480493	1.40683E-26	36.60546658	0.050157131	12.05552466	0.016849041
ENSMUSG00000090298	Sult3a2	8.446266765	0.038934427	8.867966138	1.66399E-18	1.980253378	0	2.581687003	0
ENSMUSG00000000739	Sult5a1	7.689487923	9.5427E-44	6.060138373	8.71191E-13	1.871475044	0.008961916	0.426563259	0.006268751
ENSMUSG00000026547	Tagln2	-0.837780098	3.88938E-06	-0.55469473	0.000620108	7.701469112	13.62458493	9.802572582	14.27966524
ENSMUSG00000056313	Tcim	0.666803036	0.020860006	1.058565921	0.000178412	5.582831672	3.507904778	7.047921128	3.390707446
ENSMUSG00000028011	Tdo2	-0.595562687	0.006051524	-0.566391005	0.036035291	266.5676703	404.8219216	320.6637636	478.8667424
ENSMUSG00000044468	Tent5c	0.717825751	1.54814E-05	0.660411164	0.000103981	1.95754029	1.179217012	1.809615855	1.136163506
ENSMUSG00000032440	Tgfbr2	-0.471926474	0.028926617	-0.580367473	0.004857165	4.518082419	6.284715847	4.499476516	6.634711963
ENSMUSG00000022218	Tgm1	2.71891947	9.36109E-10	1.835234037	0.002873503	7.257338295	1.107690904	4.418075145	1.216737388
ENSMUSG00000070720	Tmem200b	-1.756344829	9.81728E-10	-0.912948347	0.019671061	1.17555819	3.973738015	2.456576915	4.661841652
ENSMUSG00000041737	Tmem45b	2.622840876	0.012079423	2.519910742	0.000483261	0.108673323	0.017563588	0.10676984	0.0187862
ENSMUSG00000033177	Tmprss7	-3.140696007	0.000637124	-4.707446635	2.14473E-08	0.014028068	0.133190471	0.014488395	0.386241128
ENSMUSG00000032554	Trf	0.576585279	0.008561452	0.493086463	0.014935181	5838.968833	3946.064282	5173.328073	3669.916799
ENSMUSG00000032715	Trib3	-1.313854349	0.019123806	-1.02615444	0.042115774	2.958568762	7.312379617	2.321641281	4.743352939
ENSMUSG00000103711	Tstd1	-1.419712247	2.70871E-17	-0.923675836	0.002835228	9.872189518	26.01690304	14.70676789	27.82812763
ENSMUSG00000030428	Ttyh1	0.984112586	0.032817562	1.284997575	0.048304031	0.084971449	0.042951256	0.145802402	0.059465142
ENSMUSG00000030137	Tuba8	-2.387941991	4.50026E-08	-1.941231646	0.04926639	0.376723307	1.957513369	0.85997834	3.267789479
ENSMUSG00000090175	Ugt1a9	1.659976096	0.006608223	1.642157579	0.041151824	21.71076524	6.7170562	29.14035104	9.627167311
ENSMUSG00000035780	Ugt2a3	1.441366584	2.26628E-09	0.944021565	4.8581E-07	146.3771553	54.03674831	95.47095393	49.91170861
ENSMUSG00000019951	Uhrf1bp1l	0.390169975	0.001128094	0.44066054	0.025563034	5.960267533	4.519776928	6.020777415	4.439007346
ENSMUSG00000075543	Urad	3.729752533	0.000224321	3.83835187	5.18392E-15	55.07798323	4.325057831	26.33120007	1.826496676
ENSMUSG00000017723	Wfdc2	2.414055	4.02703E-11	1.563342673	0.001730462	3.043226729	0.57593608	1.849902229	0.624018535
ENSMUSG00000051748	Wfdc21	8.093197205	2.09218E-92	6.280709693	5.712E-23	499.1916017	1.875224763	175.4577289	2.159454411
ENSMUSG00000030170	Wnt5b	1.503310733	2.04186E-07	1.586886773	9.80807E-05	2.44846126	0.857566333	2.326469112	0.763249978
ENSMUSG00000026117	Zap70	3.437053165	8.54688E-28	2.191517307	0.000253816	4.239005374	0.394229785	3.364613045	0.735739893
ENSMUSG00000058881	Zfp516	0.378320027	0.044645386	0.585230189	0.009662245	0.756358622	0.580893235	0.862141493	0.574909262
