# Supplementary material for: GH-resistant (Laron) mice: gene therapy with a liver-specific GH receptor causes unbalanced upregulation of female-biased and growth-related genes
Source: Front Endocrinol (Lausanne). 2026 May 28;17:1808977. doi: 10.3389/fendo.2026.1808977 (PMC13253266; doi:10.3389/fendo.2026.1808977)
Supplement: Supplementary Table 2 — Expression of cancer related genes. [file DataSheet5.pdf]

Supplementary Table 2. Expression of cancer-related genes

Male & Female Mice											
		GHR+/+ vs GHR-/-		GHR+/+ vs AAV-HLP-mGHR		AAV-HLP-mGHR vs AAV-HLP-Luc		mean fpkm			
ENS ID	Gene	log2FC	p-adj	log2FC	p-adj	log2FC	p-adj	GHR+/+	GHR-/-	AAV-HLP-mGHR	AAV-HLP-Luc
ENSMUSG00000022347	A1bg	13.29125126	9.95222E-06	0.237983124	NA	14.55511917	2.94995E-62	173.2745326	0.0169753	138.7133271	0.005520957
ENSMUSG00000022178	Ajuba	1.814946037	6.20981E-23	1.099194735	1.19155E-08	1.014568436	0.00098845	2.060616128	0.587785873	0.961950202	0.470874632
ENSMUSG00000046532	Ar	3.835417448	4.28022E-07	-0.395359931	0.539450834	3.143555471	5.15966E-08	1.502527443	0.106566814	1.921949735	0.222477413
ENSMUSG00000020052	Ascl1	-1.49035351	NA	-11.96568383	5.68636E-33	11.90516121	7.32514E-33	0	0.005745738	8.012979031	0
ENSMUSG00000031963	Bmper	1.787343983	0.005415823	0.190303867	0.876100586	0.444655701	0.883530283	0.207226218	0.058854708	0.177994746	0.126243958
ENSMUSG00000016028	Celsr1	3.93972853	3.17521E-40	0.475417296	0.500063119	3.599829481	1.9855E-27	1.328654447	0.085971479	0.946595889	0.077831112
ENSMUSG00000042589	Cux2	3.11023827	0.071122385	-0.620030443	0.810495074	4.705044605	3.48193E-13	1.858983311	0.212272532	2.728068883	0.106805522
ENSMUSG00000022129	Dct	10.2890877	2.61241E-23	0.944912389	0.270922309	9.299980526	2.29221E-20	1.057740818	0	0.540247362	0
ENSMUSG00000025014	Dntt	5.295783713	1.83147E-09	0.995176257	0.242936337	5.033392474	3.46448E-15	1.757327024	0.043941516	0.857884377	0.026380904
ENSMUSG00000020122	Egfr	4.547017793	3.95766E-17	3.144587524	3.2745E-06	0.005765843	NA	49.78299851	2.209099052	5.918841767	5.597838467
ENSMUSG00000026479	Lamc2	0.204156081	0.877268222	-0.122268408	0.93822885	0.965374004	0.360783338	0.090203724	0.075937588	0.096762909	0.04836037
ENSMUSG00000054263	Lifr	1.526974765	3.02111E-12	0.051465741	0.936252501	1.604119933	1.96747E-14	33.83067566	11.76688147	32.9148817	10.77714336
ENSMUSG00000072568	Lratd2	1.148588947	0.13824598	-0.239161276	0.860351003	1.746813563	3.46072E-21	3.24803643	1.403995046	3.682443076	1.093758746
ENSMUSG00000042834	Nrep	3.719211156	7.78254E-14	1.977441399	0.000761579	1.865288358	0.009565344	7.039929642	0.543129939	1.825570981	0.493554322
ENSMUSG00000070368	Prok1	10.03699037	4.32839E-26	1.19642107	0.356182734	8.316010697	2.55242E-08	1.589731068	0	0.730369359	0.001263635
ENSMUSG00000027796	Smad9	5.524551732	2.09614E-33	2.882285193	0.009090823	2.849107661	0.076785546	2.720699381	0.060410627	0.388992021	0.052469739
ENSMUSG00000032091	Tmprss4	1.075593794	0.402205614	-2.168989804	0.013333668	3.083004624	2.30776E-08	0.088773465	0.041629881	0.385704076	0.04621499
ENSMUSG00000017723	Wfdc2	2.414055	4.02703E-11	0.730329361	0.128280361	1.563342673	0.001730462	3.043226729	0.57593608	1.849902229	0.624018535
ENSMUSG00000030170	Wnt5b	1.503310733	2.04186E-07	0.072322413	0.930551421	1.586886773	9.80807E-05	2.44846126	0.857566333	2.326469112	0.763249978
ENSMUSG00000026117	Zap70	3.437053165	8.54688E-28	0.316086947	0.462096083	2.191517307	0.000253816	4.239005374	0.394229785	3.364613045	0.735739893

Male Mice Only											
		GHR+/+ vs GHR-/-		GHR+/+ vs AAV-HLP-mGHR		AAV-HLP-mGHR vs AAV-HLP-Luc		mean fpkm			
ENS ID	Gene	log2FC	p-adj	log2FC	p-adj	log2FC	p-adj	GHR+/+	GHR-/-	AAV-HLP-mGHR	AAV-HLP-Luc
ENSMUSG00000022347	A1bg	1.984258418	NA	-13.88915116	2.163E-29	14.75345754	3.00068E-25	0.00820203	0	124.4088212	0.004216178
ENSMUSG00000022178	Ajuba	2.079828887	9.00122E-18	1.367465547	1.11152E-09	0.569020942	0.416122945	1.789843997	0.422254936	0.692602158	0.466907339
ENSMUSG00000046532	Ar	4.539844222	3.68054E-45	-1.114997794	1.43243E-12	3.807210821	0.000565147	0.946395479	0.041089512	2.044427801	0.146210944
ENSMUSG00000020052	Ascl1	NA	NA	-11.35777244	7.09607E-13	11.32698438	5.31751E-12	0	0	5.751856048	0
ENSMUSG00000031963	Bmper	0.745369595	0.502592847	-0.795073974	0.325165706	-0.518626996	0.799992799	0.075156879	0.04486891	0.130284917	0.186544479
ENSMUSG00000016028	Celsr1	3.895759878	4.77665E-11	0.299047343	0.816842246	3.681558052	3.16568E-28	1.079337437	0.072386517	0.875447306	0.068128329
ENSMUSG00000042589	Cux2	-0.268147167	0.850862021	-5.880812661	2.03491E-66	6.187861898	5.76238E-59	0.033207306	0.039806552	1.955295991	0.026547731
ENSMUSG00000022129	Dct	10.21799483	2.6314E-15	0.64074917	0.125330823	9.416323391	5.73825E-12	1.006583699	0	0.644936835	0
ENSMUSG00000025014	Dntt	5.094921691	0.00074035	0.255100347	0.901190555	4.894222949	0.09005493	1.099606834	0.032325356	0.922411737	0.030903924
ENSMUSG00000020122	Egfr	5.289396121	7.0087E-101	4.291427913	2.19387E-74	-0.995944104	0.675117068	82.20568724	2.094042804	4.182425307	8.31927699

ENSMUSG00000026479	Lamc2	-1.441438786	0.112814237	-1.053991313	0.335099157	-0.071774759	0.979910277	0.023500817	0.06364274	0.048709715	0.050734527
ENSMUSG00000054263	Lifr	1.683932074	0.001856711	0.37412666	0.709981973	1.519244818	7.88224E-10	33.16482719	10.28218566	25.48810301	8.878606617
ENSMUSG00000072568	Lratd2	-0.563482187	0.016873385	-1.918869712	8.05916E-24	1.727356382	1.82934E-12	0.876280151	1.290034634	3.303666117	0.99486364
ENSMUSG00000042834	Nrep	4.460720694	2.76841E-10	2.860244923	0.0001058	1.381671817	0.02289632	6.353395748	0.287894065	0.873622331	0.334547657
ENSMUSG00000070368	Prok1	10.33372248	1.71004E-11	3.304882981	0.000965793	6.865534334	7.72262E-05	1.984444446	0	0.200829023	0
ENSMUSG00000027796	Smad9	6.59885599	8.37357E-56	6.012052741	8.54362E-12	0.812950567	0.800603571	2.935479441	0.030332369	0.045225419	0.025541783
ENSMUSG00000032091	Tmprss4	-0.383453464	0.890032585	-4.338944799	2.65042E-09	3.54039888	1.64199E-07	0.026831633	0.034873721	0.540453818	0.045641987
ENSMUSG00000017723	Wfdc2	2.95154917	3.72221E-09	1.126472946	0.043794219	1.73427707	0.027725982	3.003124178	0.387879202	1.374193134	0.407709397
ENSMUSG00000030170	Wnt5b	1.399042783	0.000147221	0.121798007	0.952137748	1.334452162	0.391760164	2.378741294	0.901354124	2.186278823	0.862871139
ENSMUSG00000026117	Zap70	3.922855034	1.8953E-38	0.049447	0.949436838	2.282905837	0.148264668	3.163241881	0.208653159	3.054555261	0.625922698

Female Mice Only											
		GHR+/+ vs GHR-/-		GHR+/+ vs AAV-HLP-mGHR		AAV-HLP-mGHR vs AAV-HLP-Luc		mean fpkm			
ENS ID	Gene	log2FC	p-adj	log2FC	p-adj	log2FC	p-adj	GHR+/+	GHR-/-	AAV-HLP-mGHR	AAV-HLP-Luc
ENSMUSG00000022347	A1bg	13.29779326	4.01286E-09	1.174125807	0.716335789	14.40087536	8.13829E-40	346.5408632	0.0339506	153.017833	0.006825736
ENSMUSG00000022178	Ajuba	1.628387477	2.82014E-15	0.915524784	1.74494E-06	1.376056893	4.39506E-06	2.331388259	0.753316811	1.231298247	0.474841924
ENSMUSG00000046532	Ar	3.577893745	0.002654935	0.187692319	0.88877717	2.588295947	0.000533574	2.058659407	0.172044117	1.799471669	0.298743882
ENSMUSG00000020052	Ascl1	-2.681487498	NA	-12.49493316	1.71416E-22	12.40673739	2.39106E-22	0	0.011491476	10.27410201	0
ENSMUSG00000031963	Bmper	2.22166791	0.000453401	0.585561984	0.512078582	1.763378065	0.006753543	0.339295556	0.072840506	0.225704575	0.065943438
ENSMUSG00000016028	Celsr1	3.977807332	1.3318E-17	0.62548589	0.577071921	3.535836938	0.000148814	1.577971457	0.09955644	1.017744473	0.087533895
ENSMUSG00000042589	Cux2	3.255087971	0.00236915	0.067100692	0.969625486	4.22139577	8.2189E-20	3.684759316	0.384738512	3.500841774	0.187063314
ENSMUSG00000022129	Dct	10.36119603	1.33623E-08	1.340190964	0.460369907	9.09628638	5.8649E-06	1.108897937	0	0.435557889	0
ENSMUSG00000025014	Dntt	5.420849181	6.25636E-05	1.599952013	0.112745775	5.159504501	7.06254E-11	2.415047214	0.055557675	0.793357016	0.021857884
ENSMUSG00000020122	Egfr	2.897102462	1.0505E-102	1.175627565	0.053780846	1.408562552	0.035074618	17.36030978	2.3241553	7.655258227	2.876399944
ENSMUSG00000026479	Lamc2	0.808309994	0.299735648	0.105744968	0.920918723	1.6461541	0.012854243	0.15690663	0.088232435	0.144816103	0.045986213
ENSMUSG00000054263	Lifr	1.375664482	1.28162E-13	-0.231149986	0.488151165	1.667531218	4.1848E-18	34.49652413	13.25157727	40.34166039	12.6756801
ENSMUSG00000072568	Lratd2	1.880780218	2.43957E-23	0.463132094	0.132959792	1.76445894	5.52691E-15	5.61979271	1.517955457	4.061220034	1.192653851
ENSMUSG00000042834	Nrep	3.266688668	3.61731E-09	1.469957591	0.006446023	2.08679507	0.00350775	7.726463536	0.798365812	2.777519631	0.652560987
ENSMUSG00000070368	Prok1	9.608518247	2.87898E-13	-0.080015827	0.973385664	8.802760741	9.66985E-06	1.19501769	0	1.259909695	0.00252727
ENSMUSG00000027796	Smad9	4.78494972	3.56174E-12	1.769833485	3.28303E-10	3.207625824	6.96603E-19	2.505919321	0.090488884	0.732758623	0.079397695
ENSMUSG00000032091	Tmprss4	1.647940339	0.356179956	-0.602005552	0.699356809	2.316393446	0.009541393	0.150715297	0.048386041	0.230954333	0.046787992
ENSMUSG00000017723	Wfdc2	1.998190639	1.11674E-10	0.400601281	0.434801552	1.470356156	0.001718053	3.08332928	0.763992957	2.325611323	0.840327674
ENSMUSG00000030170	Wnt5b	1.62247983	6.04386E-07	0.021355994	0.975502678	1.886751839	8.65182E-13	2.518181226	0.813778542	2.4666594	0.663628817
ENSMUSG00000026117	Zap70	3.199476236	2.05483E-40	0.52665864	0.050965438	2.117857429	0.001160222	5.314768866	0.579806411	3.674670829	0.845557087
