# Supplementary material for: GH-resistant (Laron) mice: gene therapy with a liver-specific GH receptor causes unbalanced upregulation of female-biased and growth-related genes
Source: Front Endocrinol (Lausanne). 2026 May 28;17:1808977. doi: 10.3389/fendo.2026.1808977 (PMC13253266; doi:10.3389/fendo.2026.1808977)
Supplement: Supplementary Table 3 — Unaltered carbohydrate and lipid. [file DataSheet6.pdf]

Supplementary Table 3. Unaltered Expression of Carbohydrate and Lipid Metabolism-Related Genes in AAV-HLP-mGHR Treated Mice Compared to AAV-HLP-Luc Controls.

Gene	Log2 Fold Change AAV-GHR/AAV-Luc	Significance, p_{adj}	Sex
Cd36	0.272	Not Significant	Both Male and Female
Ppara	0.411	0.0691	
Pparg	-0.343	Not Significant	
Pgc1a (Ppargc1a)	-0.470	Not Significant	
Scd1	0.0972	Not Significant	
Scd2	-2.49	0.0172	
Scd3	0.533	Not Significant	
Scd4	0.258	Not Significant	
Fasn	0.439	Not Significant	
Cd36	0.410	Not Significant	Male
Ppara	0.395	Not Significant	
Pparg	-0.0867	Not Significant	
Pgc1a (Ppargc1a)	-0.559	Not Significant	
Scd1	0.0480	Not Significant	
Scd2	-2.77	0.0577	
Scd3	1.34	Not Significant	
Scd4	1.39	Not Significant	
Fasn	0.304	Not Significant	
Cd36	0.0688	Not Significant	Female
Ppara	0.427	Not Significant	
Pparg	-0.622	0.000489	
Pgc1a (Ppargc1a)	-0.408	Not Significant	
Scd1	0.197	Not Significant	
Scd2	-1.57	0.0604	
Scd3	-0.638	Not Significant	
Scd4	0.00955	Not Significant	
Fasn	0.628	Not Significant	
