# Supplementary material for: GH-resistant (Laron) mice: gene therapy with a liver-specific GH receptor causes unbalanced upregulation of female-biased and growth-related genes
Source: Front Endocrinol (Lausanne). 2026 May 28;17:1808977. doi: 10.3389/fendo.2026.1808977 (PMC13253266; doi:10.3389/fendo.2026.1808977)
Supplement: Supplementary Table 4 — Expression of carbohydrate and lipid. [file DataSheet7.pdf]

Supplementary Table 4. Expression of carbohydrate and lipid metabolism-related genes

		GHR+/+ vs GHR-/-		GHR+/+ vs AAV-HLP-mGHR		AAV-HLP-mGHR vs AAV-HLP-Luc		AAV-HLP-mGHR vs GHR-/-		mean fpkm			
ENS ID	Gene	log2FC	p-adj	log2FC	p-adj	log2FC	p-adj	log2FC	p-adj	GHR+/+	GHR-/-	AAV-HLP-mGHR	AAV-HLP-Luc
ENSMUSG00000002944	Cd36	-2.316624109	5.91558E-09	-2.341681245	2.07787E-07	0.271628722	0.812698285	0.022833866	0.987147238	14.92082444	72.05449553	72.61919804	60.4975826
ENSMUSG00000069922	Ces3a	3.334595154	3.17834E-09	1.890413923	1.80367E-06	1.510722019	8.32531E-05	1.440974959	0.053913772	505.9338047	50.53566215	137.0868819	47.8467328
ENSMUSG00000021416	Eci3	4.107489823	0.142156782	-0.55156398	0.887616264	5.330877511	1.13607E-11	4.645397329	4.07677E-07	2.671377632	0.153127492	3.673250478	0.092489209
ENSMUSG00000025153	Fasn	0.804988488	0.30772297	0.528734037	0.628320452	0.439245442	0.755269605	0.273607462	0.808760331	84.84531201	47.2660114	57.06825313	41.68525028
ENSMUSG00000015568	Lpl	-2.584953933	2.38919E-10	-0.197433306	0.853824954	-2.586961024	9.08052E-17	-2.389227989	1.34445E-11	2.355522488	13.80496203	2.685733661	15.7337342
ENSMUSG00000022383	Ppara	-0.378043011	0.122463152	-0.79861432	3.94686E-09	0.410954786	0.069119247	0.417647001	0.240306082	31.51560488	41.11491195	54.8002222	41.12548938
ENSMUSG00000000440	Pparg	-1.419547183	1.2167E-09	-0.919626341	0.001076033	-0.342853649	0.353558357	-0.502346254	0.052115717	3.648215008	9.624678535	6.815101752	8.722211261
ENSMUSG00000029167	Ppargc1a	-1.519691713	5.41115E-12	-0.875099407	0.003898527	-0.470426237	0.385033599	-0.647424713	0.019596817	1.493462114	4.178871977	2.71641299	3.750887893
ENSMUSG00000037071	Scd1	0.106272267	0.868522336	0.556881369	0.41509607	0.097183161	0.986892861	-0.452461682	0.711726699	1872.453203	1691.522469	1233.573826	1147.06625
ENSMUSG00000025203	Scd2	-3.741661515	2.29093E-18	-2.133362729	0.000528447	-2.492734674	0.017161921	-1.610821706	0.001253904	2.092729473	27.02457144	8.726671109	47.96812627
ENSMUSG00000025202	Scd3	-0.035658178	0.987034031	1.398236177	0.075923329	0.532998665	0.839277841	-1.426604948	0.316499385	0.19344328	0.190054106	0.071888603	0.049290344
ENSMUSG00000050195	Scd4	0.915581724	0.557198911	0.459986702	0.811667443	0.258144389	0.985796445	0.442820245	0.942663665	0.020709701	0.010849607	0.014799029	0.012293948
