# Supplementary material for: GH-resistant (Laron) mice: gene therapy with a liver-specific GH receptor causes unbalanced upregulation of female-biased and growth-related genes
Source: Front Endocrinol (Lausanne). 2026 May 28;17:1808977. doi: 10.3389/fendo.2026.1808977 (PMC13253266; doi:10.3389/fendo.2026.1808977)
Supplement: Supplementary Table 5 — Distribution of sex-biased genes. [file DataSheet8.pdf]

Supplementary Table 5. Distribution of sex-biased genes.

Sex	Comparison	Number of Up regulated genes			Number of Down regulated genes		
		Female-biased genes	Male-biased genes	Other genes	Female-biased genes	Male-biased genes	Other genes
Male	GHR-/- vs GHR+/+	479 (34.9%)	13 (0.9%)	879 (64.1%)	30 (1.9%)	662 (42.9%)	851 (55.2%)
Male	AAV-HLP-Luc vs AAV-HLP-mGHR	32 (12.7%)	43 (17.1%)	176 (70.1%)	86 (27.0%)	28 (8.8%)	205 (64.3%)
Female	GHR-/- vs GHR+/+	90 (13.8%)	63 (9.6%)	500 (76.6%)	126 (16.7%)	133 (17.7%)	494 (65.6%)
Female	AAV-HLP-Luc vs AAV-HLP-mGHR	26 (16.6%)	28 (17.8%)	103 (65.6%)	71 (21.8%)	44 (13.5%)	210 (64.6%)

Percentages are based on the total numbers of genes up regulated or down regulated in each row.
