# Supplementary material for: GH-resistant (Laron) mice: gene therapy with a liver-specific GH receptor causes unbalanced upregulation of female-biased and growth-related genes
Source: Front Endocrinol (Lausanne). 2026 May 28;17:1808977. doi: 10.3389/fendo.2026.1808977 (PMC13253266; doi:10.3389/fendo.2026.1808977)
Supplement: Supplementary file 9 [file DataSheet9.zip › List of Supplementary Materials.docx]

**List of Supplementary Materials**

Differentially-expressed gene (DE-gene) file is in excel format

Geneset enrichment file is in tab-delimited format

**Comparison between experimental groups (combining male and female in each group)**

| **Filename** | **Comparison Group** | **Reference Group** | **Analysis** |
| --- | --- | --- | --- |
| 1A | AAV-HLP-Luc | GHR-/- | DE-gene |
| 1B | AAV-HLP-Luc | GHR-/- | Geneset enrichment |
| 2A | AAV-HLP-mGHR | AAV-HLP-Luc | DE-gene |
| 2B | AAV-HLP-mGHR | AAV-HLP-Luc | Geneset enrichment |
| 3A | GHR+/+ | AAV-HLP-mGHR | DE-gene |
| 3B | GHR+/+ | AAV-HLP-mGHR | Geneset enrichment |
| 4A | GHR+/+ | GHR-/- | DE-gene |
| 4B | GHR+/+ | GHR-/- | Geneset enrichment |

**Gender-specific genes in wild-type mice**

| **Filename** | **Comparison Group** | **Reference Group** | **Analysis** |
| --- | --- | --- | --- |
| 5A | GHR+/+ Male | GHR+/+ Female | DE-gene |
| 5B | GHR+/+ Male | GHR+/+ Female | Geneset enrichment |

**Comparison between experimental groups by gender**

| **Filename** | **Comparison Group** | **Reference Group** | **Analysis** |
| --- | --- | --- | --- |
| 6A | AAV-HLP-mGHR Male | AAV-HLP-Luc Male | DE-gene |
| 6B | AAV-HLP-mGHR Male | AAV-HLP-Luc Male | Geneset enrichment |
| 7A | GHR+/+ Male | GHR-/- Male | DE-gene |
| 7B | GHR+/+ Male | GHR-/- Male | Geneset enrichment |
| 8A | GHR+/+ Male | AAV-HLP-mGHR Male | DE-gene |
| 8B | GHR+/+ Male | AAV-HLP-mGHR Male | Geneset enrichment |
| 9A | AAV-HLP-mGHR Female | AAV-HLP-Luc Female | DE-gene |
| 9B | AAV-HLP-mGHR Female | AAV-HLP-Luc Female | Geneset enrichment |
| 10A | GHR+/+ Female | GHR-/- Female | DE-gene |
| 10B | GHR+/+ Female | GHR-/- Female | Geneset enrichment |
| 11A | GHR+/+ Female | AAV-HLP-mGHR Female | DE-gene |
| 11B | GHR+/+ Female | AAV-HLP-mGHR Female | Geneset enrichment |

**Gene expression matrix and annotation of experimental design**

12A Gene expression matrix (raw counts)

12B Gene expression matrix (fpkm normalized)

12C Annotation file
